# Phaeosphamides A and B, Cytotoxic Cyclodecadepsipeptides from the Mangrove-Derived Fungus *Phaeosphaeriopsis* sp. S296

**DOI:** 10.3390/md20100591

**Published:** 2022-09-21

**Authors:** Siwen Niu, Jianlin He, Shuhuan Huang, Shouyuan Wu, Ling Zeng, Juan Wang, Bihong Hong, Ziming Chen

**Affiliations:** 1 School of Chemistry and Chemical Engineering, Lingnan Normal University, Zhanjiang 524048, China; 2 Technology Innovation Center for Exploitation of Marine Biological Resources, Third Institute of Oceanography, Ministry of Natural Resources, Xiamen 361005, China; 3 Mangrove Institute, Lingnan Normal University, Zhanjiang 524048, China

**Keywords:** *Phaeosphaeriopsis* sp., cyclodecadepsipeptides, marine natural products, cytotoxic activity

## Abstract

Chemical examination of the fermented broth of the mangrove-derived fungus *Phaeosphaeriopsis* sp. S296 resulted in the isolation of two new cyclodecadepsipeptides, namely phaeosphamides A (**1**) and B (**2**), as well as one known congener Sch 217048 (**3**). The structures of new metabolites, including absolute configurations, were established on the basis of extensive spectroscopic data analyses, chemical conversion, and Marfey’s method. The 2-hydroxy-3-methylpentanoic acid (Hmp) moiety and pipecolic acid (Pip) unit in structures were rarely discovered in nature. Interestingly, compounds **1**–**3** are examples of peptides discovered from the fungal genus *Phaeosphaeriopsis* for the first time. All identified compounds were evaluated for their cytotoxicity against five tumor cell lines of AGS, BEL-7402, HepG2, B16, and BIU87. Among them, compound **1** showed inhibitory activities against these tumor cell lines with IC_50_ values ranging from 5.14 to 66.38 μM. A further mechanistic investigation found that **1** arrested AGS cells in the G2 phase and induced their apoptosis in a dose-dependent manner.

## 1. Introduction

Cyclic depsipeptides are a class of cyclopeptides in which one or more amide groups were replaced by lactone bond in the core ring, exhibiting a wide range of biological effects, such as cytotoxic, antimicrobial, antimalarial, and anti-HIV activities [1,2,3,4]. The structural variety of the peptides is attributed to the incorporation of distinct amino acid residues and hydroxylated carboxylic acids, in addition to unusual amino acid residues and various chemical modifications [5,6,7]. Multiple cyclic depsipeptides have been discovered in bacteria, fungi, algae, sponges, and other organisms. Some of the congeners possessed pharmaceutical uses, such as romidepsin obtained from *Chromobacterium violaceum* bearing the histone deacetylase inhibitory effect [8]; daptomycin, isolated from *Streptomyces roseosporus*, has been used in the treatment of skin and skin-structure infections [9]; plusbacin A_3_, isolated from *Pseudomonas* sp., exhibited inhibitory effects against vancomycin-resistant bacteria [10]; and emodepside, a semisynthetic derivative of PF1022A, is applied as an anthelmintic agent [11].

**Figure 1:**
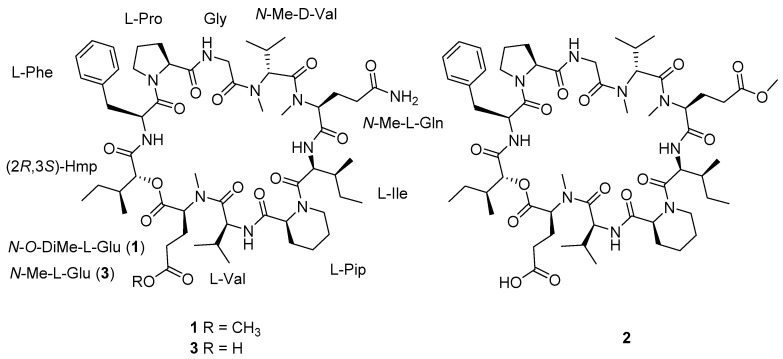
Chemical structures of compounds **1**–**3** from *Phaeosphaeriopsis* sp. S296.

Cyclodecadepsipeptides, a subclass of cyclic depsipeptides, are usually constructed by nine amino acid residues and a hydroxylated carboxylic acid. To date, a total of 21 cyclodecadepsipeptides have been reported from nature [12,13,14,15,16,17,18]. Among them, only three naturally occurring analogs bearing a rare pipecolic acid residue, namely Sch 217048 [19], Sch 218157 [20], and Sch 378167 [18], were exclusively found from the fungi. The structural novelty and significant pharmaceutical activities of cyclodecadepsipeptides have attracted considerable attention from natural product chemists and pharmacologists. As part of our ongoing research to discover new and/or bioactive secondary metabolites from marine-derived fungi [21,22,23,24], a chemical investigation of the EtOAc extract of fermented broth of the mangrove-derived fungus *Phaeosphaeriopsis* sp. S296 led to the isolation of two new cyclodecadepsipeptides, namely phaeosphamides A (**1**) and B (**2**), together with one known congener Sch 217048 (**3**) (Figure 1). The new structures were assigned on the basis of the spectroscopic data analyses, chemical conversion, and Marfey’s method. Compounds **1** and **2** represent the fourth examples of cyclodecadepsipeptides bearing the rare pipecolic acid residue in the core ring. Additionally, peptides **1** and **2** were first discovered in the genus *Phaeosphaeriopsis*. Cytotoxicity evaluation revealed that **1** exhibited inhibitory activities against five tumor cell lines of AGS, BEL-7402, HepG2, B16, and BIU87. Further investigation discovered that **1** arrested cell cycle in the G2 phase and induced apoptosis in AGS tumor cells in a dose-dependent manner. Herein, the isolation, structural elucidation and cytotoxicity of compounds **1**–**3** are presented.

## 2. Results and Discussion

**Figure 2:**
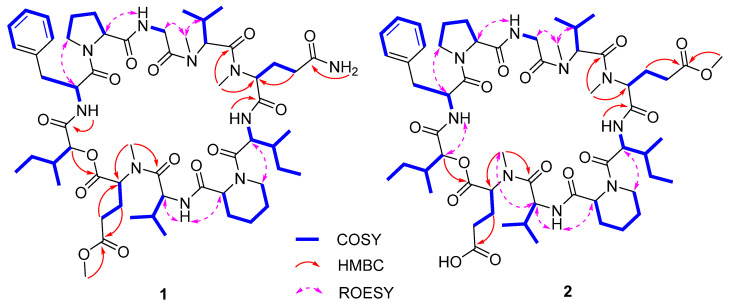
Selected COSY, HMBC, and REOSY correlations of **1** and **2**.

Compound **1** was purified as a white powder. The molecular formula of **1** was assigned to be C_58_H_90_N_10_O_14_ on the basis of a protonated ion peak at *m*/*z* 1151.6706 [M + H]^+^ (calcd for C_58_H_91_N_10_O_14_, 1151.6711) in the HRESIMS spectrum, in association with the ^13^C NMR data, requiring 19 indices of hydrogen deficiency. From the ^1^H and ^13^C NMR spectra, the presence of six exchangeable protons (*δ*_H_ 9.23, 8.57, 8.29, 8.06, 7.65, and 6.74), a series of *α* protons resonances at *δ*_H_ 4.37–5.63, four *N*/*O*-methyls (*δ*_H_ 3.00, 3.19, 3.52, and 3.62), and 12 carbonyl carbon signals (*δ*_C_ 169.1, 169.6, 170.0, 170.7, 171.1, 171.2, 171.4, 171.4, 172.8, 173.2, 173.3, and 174.8) indicated that **1** was a peptide structure. The NMR spectroscopic data of **1** were similar to those of the co-isolated cyclodepsipeptide Sch 217048 (**3**) [19], indicating that they were structurally related derivatives except for an additional methoxy signals (*δ*_H_ 3.62, *δ*_C_ 51.5) in **1**. Interpretation of 2D (HSQC, COSY, and HMBC spectra) NMR data of **1** determined the presence of nine proteinogenic amino acids: phenylalanine (Phe), proline (Pro), glycine (Gly), *N*-methylvaline (*N*-Me Val), *N*-methylglutamine (*N*-Me Gln), isoleucine (Ile), pipecolic acid (Pip), valine (Val), and *N*-*O*-dimethylglutamic acid (*N*-*O*-DiMe Glu). In addition, the 2-hydroxy-3-methylpentanoic acid (Hmp) unit was elucidated by the COSY cross-peaks of H-2 (*δ*_H_ 5.52)/H-3 (*δ*_H_ 2.31)/3-Me (*δ*_H_ 0.88)/H-3/H_2_-4 (*δ*_H_ 1.15)/H_3_-5 (*δ*_H_ 0.70), as well as the HMBC interaction from H-2 to C-1 (*δ*_C_ 169.1). Comparison of the amino acid residues between **1** and **3** discovered that the sole difference was the presence of *N*-*O*-DiMe Glu in **1** instead of *N*-Me Glu in **3**, as evidenced by the COSY correlations of Hmp from H_2_-3 (*δ*_H_ 2.85, 2.68) to H-2 (*δ*_H_ 4.37) and H_2_-4 (*δ*_H_ 2.70) as well as the HMBC cross-peaks from H_2_-3 to C-5 (*δ*_C_ 173.2) and from the additional methoxy protons (*δ*_H_ 3.62) to C-5 (Figure 2). The assembly sequence of nine amino acids and Hmp was corroborated to be the same as **3** according to the HMBC and ROESY correlations (Figure 2). The HMBC correlation from NH (*δ*_H_ 8.06) of Phe to the ester carbonyl carbon (*δ*_C_ 169.1) of Hmp, as well as the ROESY cross-peaks from H-2 (*δ*_H_ 5.34) of Phe to H_2_-5 (*δ*_H_ 3.87) of Pro, from NH (*δ*_H_ 8.57) of Gly to H-2 (*δ*_H_ 4.89) of Pro, and from *N*-Me (*δ*_H_ 3.19) of *N*-Me Val to H_2_-2b (*δ*_H_ 4.77) of Gly/H-3 (*δ*_H_ 2.52) of *N*-Me Val revealed the following sequence: Hmp-Phe-Pro-Gly-*N*-Me Val. According to the HMBC correlations from *N*-Me (*δ*_H_ 3.00) of *N*-Me Gln to carbonyl carbon (*δ*_C_ 170.7) of *N*-Me Val and from NH (*δ*_H_ 6.74) of Ile to carbonyl carbon (*δ*_C_ 169.6) of *N*-Me Gln further extended the sequence to be *N*-Me Val-*N*-Me Gln-Ile. Furthermore, the additional ROESY correlations (Figure S15) from H-2 (*δ*_H_ 5.22) of Ile to H_2_-6 (*δ*_H_ 3.77) of Pip, from NH (*δ*_H_ 9.23) of Val to H-2 (*δ*_H_ 5.54) of Pip, from *N*-Me (*δ*_H_ 3.52) of *N*-*O*-DiMe Glu to H-2 (*δ*_H_ 4.94) of Val established the Ile-Pip-Val-*N*-*O*-DiMe Glu sequence fragment. Finally, the ester linkage between Hmp and *N*-*O*-DiMe Glu was determined by the HMBC correlation from H-2 of Hmp to carbonyl carbon (C-1, *δ*_C_ 170.0) of *N*-*O*-DiMe Glu to construct a cyclodecadepsipeptide type structure.

The absolute configuration of **1** was resolved on the basis of the chemical conversion. According to the structural relationship between **1** and **3**, compound **3** was methylated with (trimethylsilyl) diazomethane to produce methyl ester derivative **3a** [25,26]. The identical ^1^H NMR data between **3a** and **1** measured in pyridine-*d*_5_ at 600 MHz (Figure S34), in association with quite similar specific rotation data between **3a** ([*α*${]}_{D}^{25}$ −102, MeOH) and **1** ([*α*${]}_{D}^{25}$ −98, MeOH) indicated the same structure of both compounds. Consequently, the amino acids and Hmp configurations of **1,** as well as their sequence order, were elucidated to be the same as those of **3**. Accordingly, the structure of **1** was established to be cyclo-(*N*-*O*-DiMe L-Glu-L-Val-L-Pip-L-Ile-*N*-Me L-Gln-*N*-Me D-Val-Gly-L-Pro-L-Phe-(2*R*,3*S*)-Hmp), and given the trivial name of phaeosphamide A.

Phaeosphamide B (**2**) was obtained as a white powder, and its molecular formula was determined to be C_58_H_89_N_9_O_15_ by the HRESIMS spectrum at *m/z* 1152.6538, [M + H]^+^ (calcd for C_58_H_90_N_9_O_15_, 1152.6551) and ^13^C NMR data. The ^1^H NMR spectrum of **2** exhibited three *N*-methylamide groups (*δ*_H_ 3.00, 3.19, and 3.55), one methoxy (*δ*_H_ 3.65), eight shielded methyl groups (*δ*_H_ 0.72, 0.78, 0.89, 0.93, 0.95, 1.03, 1.08, and 1.13), four exchangeable protons (*δ*_H_ 6.72, 8.09, 8.57, and 9.26), and several typical signals of amino acid *α* protons ranging from *δ*_H_ 4.44–5.52. The ^13^C NMR spectrum displayed 12 carbonyl carbons (*δ*_C_ 169.1, 169.3, 170.1, 170.5, 171.1, 171.2, 171.3, 171.3, 172.7, 172.9, 173.3, and 175.4), six aromatic carbons, and a series of aliphatic sp^3^ carbons. The aforementioned NMR data were similar to those of **3,** except that two exchangeable amide protons in **3** were replaced by one methoxy (*δ*_H_ 3.65; *δ*_C_ 51.5) in **2**. The above observation, in association with the molecular formula of **2,** revealed the presence of the methyl *N*-Me Glu in **2** instead of *N*-Me Gln in **3**. The assumption was authenticated on the basis of the COSY correlations of *N*-*O*-DiMe Glu of H-2 (*δ*_H_ 5.45)/H_2_-3 (*δ*_H_ 2.19, 2.69)/H_2_-4 (*δ*_H_ 2.60) as well as the HMBC cross-peaks from H_2_-3 and OCH_3_ (*δ*_H_ 3.65) to C-5 (*δ*_C_ 172.9). The linkage sequence of nine amino acids and Hmp was elucidated to be identical to **3** according to the HMBC and ROESY experiments (Figure 2). The configurations of the amino acid residues of **2** were resolved by the advanced Marfey’s method [27]. Compounds **2** and **1** were separately hydrolyzed in 6 M HCl (110 °C, 24 h) and then derivatized with 1-fluoro-2,4-dinitrophenyl-5-L-alaninamide (L-FDAA). The HPLC chromatogram of derivatives of **2** was completely consistent with that of **1,** indicating the identical amino acid configurations in both compounds (Figure S35). The conclusion was further confirmed by the same amino acid residues in **2** and **1** after acid hydrolysis since the *N*-Me Gln residue in **1** has been converted into *N*-Me Glu [19,28]. Therefore, **2** was determined as cyclo-(*N*-Me L-Glu-L-Val-L-Pip-L-Ile-*N*-*O*-DiMe L-Glu-*N*-Me D-Val-Gly-L-Pro-L-Phe-(2*R*,3*S*)-Hmp).

**Figure 3:**
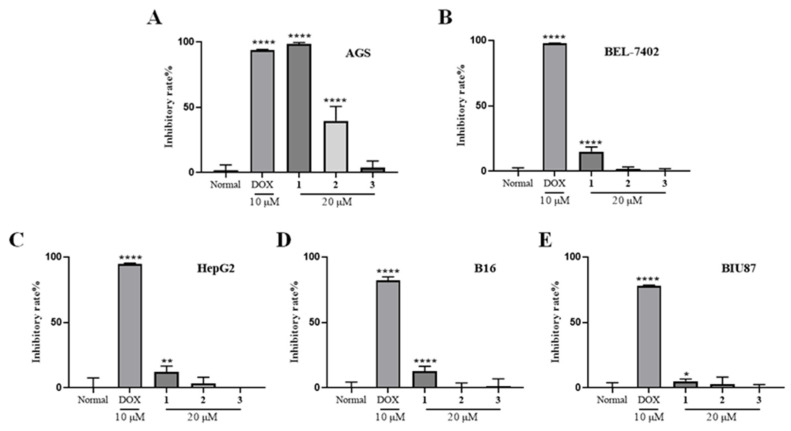
Cytotoxicity of compounds **1**–**3** against cancer cell lines of AGS (**A**), BEL-7402 (**B**), HepG2 (**C**), B16 (**D**), and BIU87 (**E**). * *p* < 0.05, ** *p* < 0.01, **** *p* < 0.0001 vs. Normal.

**Figure 4:**
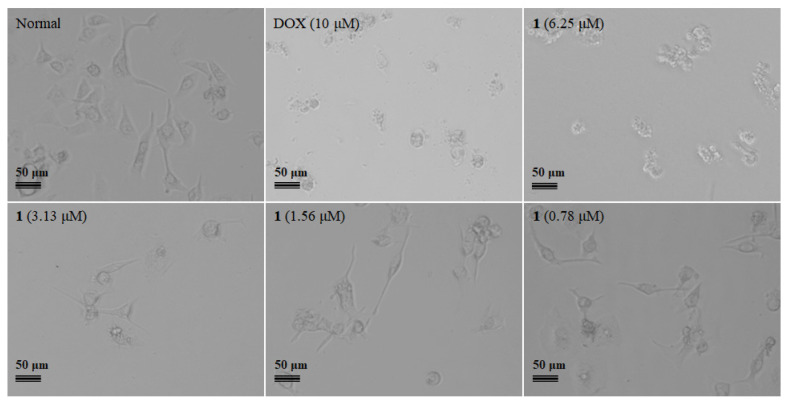
Compound **1** inhibited AGS cell proliferation with the concentration range from 0 to 10 μM.

The inhibitory effects of compounds **1**–**3** (20 μM) against five cancer lines, including AGS (human gastric adenocarcinoma cells), BEL-7402 (human hepatocarcinoma cells), HepG2 (human hepatocellular carcinomas cells), B16 (mouse melanoma cells), and BIU87 (human bladder cancer cells) were evaluated, and docetaxel (DOX) was used as the positive control. Among them, compound **1** showed the selective inhibitory effects against AGS cells at the concentration of 20 μM, and the effect is equivalent to the positive drug DOX at 10 μM (Figure 3). Further study indicated that compound **1** inhibited the proliferation of the AGS cells with an IC_50_ value of 5.14 μM (Figure 4). A primary structure-activity relationship analysis discovered that the methoxy group located at C-5 of *N*-Me Glu in **1** was critical for the cytotoxic effects.

**Figure 5:**
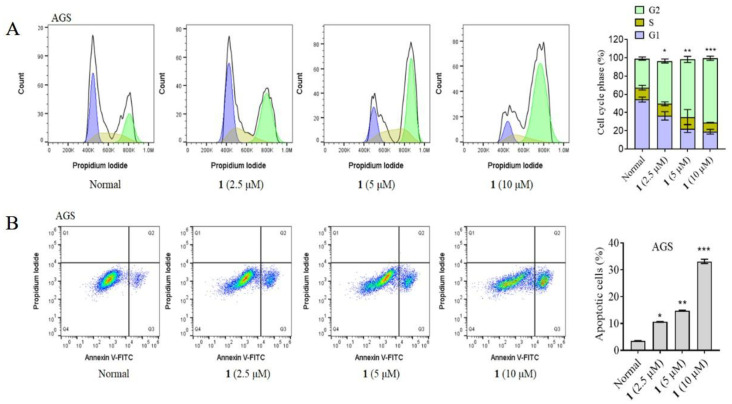
(**A**) Compound **1** induces cell cycle arrest of AGS cells at G2 phase. (**B**) Compound **1** induces apoptosis of AGS cells. Cells were treated with **1** at the concentration range from 0 to 10 μM for 24 h. * *p* < 0.05, ** *p* < 0.01, *** *p* < 0.001 vs. Normal.

As is known to all, the cell cycle can be divided into interphase and mitotic phase, while interphase is often further divided into G1, S, and G2 phases. Physical or chemical damages can trigger cell cycle arrest. To reveal the underlying anticancer mechanism of **1**, the cell cycle distribution of AGS cells after treatment of **1** was investigated. The flow cytometry analysis found that **1** arrested AGS cells at the G2 phase in a dose-dependent manner (Figure 5A). Furthermore, whether compound **1** induced the apoptosis of AGS cells was also investigated by flow cytometry assay. As shown by the Annexin V-FITC/PI double staining results in Figure 5B, the percentage of apoptotic cells was dose-dependently increased after exposure to **1** with the concentration range from 2.5 to 10 μM. The above results indicated that **1** induced apoptosis of AGS cells in a dose-dependent manner.

## 3. Materials and Methods

### 3.1. General Experimental Procedures

Optical rotation data were recorded in methanol on the Anton Paar MCP 500 automatic polarimeter. Ultraviolet data were measured in methanol from 200 to 400 nm on a Shimadzu UV-1800 spectrophotometer (Kyoto, Japan). The NMR spectra were measured on Bruker Avance 400 and 600 MHz spectrometers (Billerica, MA, USA) with TMS an internal standard. Chemical shifts (*δ*) were expressed on parts per million (ppm) scale reference to the solvent signals of pyridine-*d*_5_, and coupling constants (*J*) were expressed in Hz. Structural elucidations were performed by the additional HSQC, COSY, HMBC, and ROESY experiments. HRESIMS data were recorded on the Thermo Fisher Q Exactive mass spectrometer (Thermo, Waltham, MA, USA). HPLC analyses were performed on Waters ACQUITY Arc with a Cosmosil 5C18-MS-II (4.6 mm × 250 mm, 5 μm) column. Sephadex LH-20 (Pharmacia Biotech AB, Uppsala, Sweden) and silica gel (100–200 and 200–300 mesh, Yantai Jiangyou Silica Gel Development Co., Ltd., Yantai, China) were used for column chromatography (CC). Semi-preparative HPLC was carried out on a Shimadzu LC-16A liquid chromatography system equipped with UV/VIS variable wavelength detector, and the YMC-Pack ODS-A column (10 mm × 250 mm, 5 μm) was used for the isolation. The TLC analyses were performed on the precoated GF254 silica gel plates (Qingdao Haiyang Chemical Co., Ltd., Qingdao, China), and then the spots were visualized by heating silica gel plates sprayed with a sulfuric acid-vanillin chromogenic reagent. All solvents used in CC were analytical grade (Shanghai Chemical Reagents Co., Ltd., Shanghai, China) and used for HPLC were HPLC grade (Sigma-Aldrich, St. Louis, MI, USA).

### 3.2. Fungal Material and Identification

The producing fungus was obtained from the rhizosphere sediment of a mangrove plant *Bruguiera gymnorhiza*, which was collected from Techeng Isle, Zhanjiang, Guangdong Province, China, in July 2017. The fungus was identified as *Phaeosphaeriopsis* sp. S296 on the basis of the internal transcribed spacer (ITS) rDNA gene sequence, which exhibited a 100% identity with that of *Phaeosphaeriopsis* sp. Esf-30 (GenBank accession number OK242772). The ITS gene sequence was submitted to the GenBank database and assigned the accession number OP167982. The fungus was deposited in the Technology Innovation Center for Exploitation of Marine Biological Resources, Third Institute of Oceanography, Ministry of Natural Resources, China.

### 3.3. Fermentation, Extraction, and Isolation

The fungus was cultured on a PDA medium at 25 °C for 4 days. Then, the fresh mycelia and spores were inoculated into a PDB medium on a rotary shaker at 180 rpm at 25 °C for 4 days to prepare seed cultures. Scale-up fermentation was carried out in 500 mL Fernbach flasks (×30), each containing 80 g of rice and 100 mL of sea water. The rice and sea water mixtures were soaked overnight before autoclaving at 15 psi for 30 min. Then, after cooling to room temperature, each flask was inoculated with 3.0 mL of the seed cultures and then cultured at 28 °C in static conditions for 40 days.

The fermented broth was exhaustively extracted with ethyl acetate (EtOAc) for three times. The combined EtOAc was evaporated under reduced pressure to obtain the organic extract (35.2 g). The EtOAc extract was subjected to CC on silica gel eluting with increasing polarity from CH_2_Cl_2_ to MeOH (1:0~0:1) to yield six fractions (A~F). Fraction C (5.5 g) was chromatographed via ODS CC with MeOH/H_2_O gradient elution (30%~100%) to obtain fifteen subfractions (SFC1–SFC15). Subfraction SFC3 (950.0 mg) was fractionated on the basis of the CC over silica gel, eluting with petroleum ether/Me_2_CO (from 10:1 to 3:1) to yield five fractions (SFC3-1~SFC3-5). Subfraction SFC3-2 was purified by sephadex LH-20 (MeOH) to obtain **3** (26.1 mg). Compound **2** (32.1 mg) was obtained from subfraction SFC4 (241.2 mg) by CC over sephadex LH-20 (MeOH) and preparative HPLC (MeOH/H_2_O, 2:3). Subfraction SFC5 (133.4 mg) was subjected to silica gel CC eluting with petroleum ether/Me_2_CO (4:1) to yield four subfractions (SFC5-1~SFC5-4). Subfraction SFC5-2 was purified by preparative HPLC with a mobile phase of 45% MeOH in H_2_O to obtain **1** (15.8 mg).

Phaeosphamide A (**1**): white powder; [*α*${]}_{D}^{25}$ −98 (*c* 1.0, MeOH); UV (MeOH) *λ*_max_ (log *ε*) 210 (1.39) nm; ^1^H and ^13^C NMR data, see Table 1; HRESIMS *m*/*z* 1151.6706 [M + H]^+^ (calcd for C_58_H_91_N_10_O_14_, 1151.6711).

Phaeosphamide B (**2**): white powder; [*α*${]}_{D}^{25}$ −96 (*c* 1.0, MeOH); UV (MeOH) *λ*_max_ (log *ε*) 213 (2.01) nm; ^1^H and ^13^C NMR data, see Table 1; HRESIMS *m*/*z* 1152.6538 [M + H]^+^ (calcd for C_58_H_90_N_9_O_15_, 1152.6551).

### 3.4. Reaction of ***3*** with TMSCHN_2_ to Yield Methylated Derivative ***3a***

Compound **3** (5.0 mg) was dissolved in 1 mL of toluene/methanol (3:1) and then stirred at room temperature. (Trimethylsilyl) diazomethane (TMSCHN_2_, 5 µL) was slowly added to the mixture. After completion of the reaction, as indicated by TLC analysis, the mixtures were evaporated and then chromatographed on a semi-preparative HPLC eluting with 90% MeOH in H_2_O to yield **3a** (4.2 mg). The ^1^H NMR spectrum of **3a** measured in pyridine-*d*_5_ at 600 MHz was indistinguishable from that of **1** (**1** was remeasured with pyridine-*d*_5_ at 600 MHz).

### 3.5. Marfey’s Analysis of the Acid Hydrolysate of ***1*** and ***2***

Approximately 0.5 mg of **1** and **2** were separately hydrolyzed with 6 N HCl (600 µL) for 24 h at 110 °C. The hydrolyzed product was concentrated to dryness and then re-dissolved in H_2_O (50 µL). Subsequently, 1 N NaHCO_3_ (20 µL) and 1% FDAA solution in acetone (100 μL) were added to the aqueous hydrolysate and then heated at 40 °C for 40 min. The reaction was quenched with 1 N HCl (20 μL) and then evaporated to dryness under reduced pressure and subsequently dissolved in MeCN/H_2_O (8:1, 800 µL). The prepared derivatives of **1** and **2** were separately subjected to reversed-phase HPLC analysis (Waters ACQUITY Arc) equipped with a Cosmosil 5C18-MS-II (4.6 mm × 250 mm, 5 μm) column and eluted with a linear gradient starting at 20% solvent B increasing to 60% over 60 min (solvent A: 0.1% formic acid in H_2_O; solvent B: MeCN containing 0.1% formic acid) at a flow rate of 1 mL/min. The detection wavelength was performed with 340 nm. 

### 3.6. Cell Culture

AGS, HepG2, and B16 cell lines (Cell Bank of the Chinese Academy of Sciences, Shanghai, China) as well as BIU87 and BEL-7402 (Jining Shiye, Shanghai, China) were cultured in DMEM (Gibco, Carlsbad, CA, USA) or RPMI 1640 medium (Gibco) medium with 10% fetal bovine serum (FBS; Gibco). Cells were incubated at 37 °C in 5% CO_2_. Exponentially growing cells were used for experiments.

### 3.7. Cell Proliferation Assay

Cells were seeded into a 96-well plate at 5 × 10^3^ cells per well and cultured overnight. Then, **1**–**3** were added at designated concentrations (n = 6) and cultured for 72 h. Doxorubicin (DOX; MCE, Monmouth Junction, NJ, USA) was used as the positive control. DMSO was used as vehicle control. The digital images of the cells were taken with a Nikon E80i microscope (Nikon, Tokyo, Japan). After that, the original cultured medium was removed, and a 100 μL new medium with 10% Cell Counting Kit-8 (CCK8; MCE) was added to each well and cultured for 2 h. The absorbance at 450 nm was detected with a microplate reader (Tecan Sunrise, TECAN Deutschland GmbH, Crailsheim, Germany), and the inhibitory rate was calculated according to the manufacturer’s instructions. The IC_50_ value was calculated with Prism 8 software (Graphpad, San Diego, CA, USA) from the nonlinear regression of the percentage of inhibition versus the log10 inhibitor concentration.

### 3.8. Analysis of Cell Cycle Distribution 

Cells were cultured in a 6-well plate at about 1 × 10^6^ cells per well and treated with **1** at designated concentrations for 24 h. Then the cells were stained with a Cell Cycle Detection Kit (#KGA512, Nanjing KeyGen Biotech, Nanjing, China) according to the manufacturer’s instructions and detected with a flow cytometry under 488 nm (FACS LSR II system, BD BioSciences, Franklin Lakes, New Jersey, USA). The percentages of the cells within different cell cycle compartments were analyzed with FlowJo software version 10.6 (FlowJo LLC, Ashland, OR, USA).

### 3.9. Detection of Cell Apoptosis

Cells were seeded into a 6-well plate at about 1 × 10^6^ cells per well and treated with **1** at designated concentrations for 24 h. The assay was carried out by flow cytometry (FACS LSR II system) using Annexin V-FITC Apoptosis Detection Kit (#KGA105, Nanjing KeyGen Biotech) according to the manufacturer’s instructions. The percentages of the annexin-positive cells were analyzed with FlowJo software version 10.6 (FlowJo LLC).

## 4. Conclusions

In summary, two new cyclodecadepsipeptides, phaeosphamides A and B (**1** and **2**), together with one known analog Sch 217048 (**3**), were isolated from the mangrove-derived fungus *Phaeosphaeriopsis* sp. S296. The new structures were determined by spectroscopic analysis, in association with chemical conversion, and Marfey’s analysis of acid hydrolysates. Compounds **1**–**3** were the first representatives of peptides found in the fungal genus *Phaeosphaeriopsis*. Additionally, compounds **1**–**3** were evaluated for their inhibitory activities against five tumor cell lines of AGS, BEL-7402, HepG2, B16, and BIU87. Among them, **1** exhibited a selective inhibitory effect towards AGS cells with the IC_50_ value of 5.14 µM. Further mechanism study discovered that **1** arrested AGS cells in the G2 phase and induced apoptosis in a dose-dependent manner, suggesting that **1** can be considered a promising lead compound for the therapeutic of gastric adenocarcinoma.

## Figures and Tables

**Table 1:** ^1^H (400 MHz) and ^13^C (100 MHz) NMR data for **1** and **2** in pyridine-*d*_5_.

1				2			
Unit	No.	*δ*_C_	*δ*_H_	Unit	No.	*δ*_C_	*δ*_H_
*N-O*-DiMe Glu	1	170.0, C		*N*-Me Glu	1	170.1, C	
	2	62.8, CH	4.37, dd (8.4, 4.6)		2	63.0, CH	4.44, dd (8.4, 4.7)
	3	24.7, CH_2_	2.85, m; 2.67, m		3	24.9, CH_2_	2.95, m; 2.80, m
	4	30.5, CH_2_	2.70, m		4	30.9, CH_2_	2.82, m
	5	173.2, C			5	175.4, C	
	*N*Me	38.9, CH_3_	3.52, s		*N*Me	38.9, CH_3_	3.55, s
	*O*Me	51.5, CH_3_	3.62, s				
Val	1	173.3, C		Val	1	173.3, C	
	2	55.1, CH	4.94, t (10.0)		2	55.1, CH	4.95, t (10.1)
	3	31.7, CH	2.40, m		3	31.7, CH	2.41, m
	4	18.2, CH_3_	1.09, d (6.5)		4	18.2, CH_3_	1.08, d (6.6)
	5	19.3, CH_3_	0.89, d (6.5)		5	19.4, CH_3_	0.93, d (6.6)
	NH		9.23, d (10.0)		NH		9.26, d (10.1)
Pip	1	172.8, C		Pip	1	172.7, C	
	2	52.9, CH	5.54, br. d (4.3)		2	52.9, CH	5.51, overlapped
	3	28.0, CH_2_	2.14, m; 1.55, m		3	28.0, CH_2_	2.15, m; 1.51, m
	4	19.9, CH_2_	1.35, m		4	19.9, CH_2_	1.35, m
	5	24.8, CH_2_	1.59, m; 1.08, m		5	24.7, CH_2_	1.58, m; 1.05, m
	6	43.8, CH_2_	3.77, m		6	43.7, CH_2_	3.74, m
Ile	1	171.2, ^b^ C		Ile	1	171.2, ^d^ C	
	2	53.7, CH	5.22, br. d (8.2)		2	53.7, CH	5.20, br. d (8.0)
	3	36.8, CH	2.05, m		3	36.7, CH	2.04, m
	4	23.0, CH_2_	1.59, m; 1.03, m		4	22.9, CH_2_	1.57, m; 1.01, m
	5	11.7, CH_3_	0.95, t (6.8)		5	11.7, CH_3_	0.95, t (6.8)
	3-Me	16.5, CH_3_	1.11, d (6.7)		3-Me	16.5, CH_3_	1.13, d (6.7)
	NH		6.74, d (8.2)		NH		6.72, d (8.3)
*N*-Me Gln	1	169.6, C		*N-O*-DiMe Glu	1	169.3, C	
	2	59.9, CH	5.60, t (6.7)		2	59.4, CH	5.45, t (7.2)
	3	25.3, CH_2_	2.76, m; 2.40, m		3	24.4, CH_2_	2.69, m; 2.19, m
	4	32.0, CH_2_	2.64, m		4	30.3, CH_2_	2.60, m
	5	174.8, C			5	172.9, C	
	*N*Me	29.7, CH_3_	3.00, s		*N*Me	29.7, CH_3_	3.00, s
	5-NH_2_		7.65, s; 8.29, s		*O*Me	51.5, CH_3_	3.65, s
*N*-Me Val	1	170.7, C		*N*-Me Val	1	170.5, C	
	2	57.9, CH	5.63, d (10.5)		2	57.9, CH	5.52, overlapped
	3	28.0, CH	2.52, m		3	28.1, CH	2.55, m
	4	18.1, CH_3_	0.73, d (6.5)		4	18.1, CH_3_	0.78, d (6.5)
	5	19.3, CH_3_	0.99, d (6.5)		5	19.2, CH_3_	1.03, d (6.5)
	*N*Me	28.7, CH_3_	3.19, s		*N*Me	28.7, CH_3_	3.19, s
Gly	1	171.1, ^b^ C		Gly	1	171.1, ^d^ C	
	2	42.5, CH_2_	5.08, dd (17.4, 7.8)4.77, d (17.4)		2	42.5, CH_2_	5.09, dd (17.4, 7.9)4.78, d (17.4)
	NH		8.57, d (7.8)		NH		8.57. d (7.9)
Pro	1	171.4, ^a^ C		Pro	1	171.3, ^c^ C	
	2	60.9, CH	4.89, dd (7.2, 5.1)		2	60.8, CH	4.89, dd (7.3, 5.1)
	3	29.6, CH_2_	2.11, m; 2.03, m		3	29.5, CH_2_	2.10, m; 1.99, m
	4	25.2, CH_2_	2.09, m; 1.69, m		4	25.2, CH_2_	2.08, m; 1.69, m
	5	47.6, CH_2_	3.87, m		5	47.5, CH_2_	3.85, m
Phe	1	171.4, ^a^ C		Phe	1	171.3, ^c^ C	
	2	53.4, CH	5.34, t (10.6)		2	53.4, CH	5.35, td (8.8, 4.0)
	3	37.6, CH_2_	3.52, overlapped		3	37.6, CH_2_	3.53, overlapped
	4	138.2, C			4	138.2, C	
	5, 9	129.9, CH	7.67, d (7.5)		5, 9	129.9, CH	7.69, d (7.5)
	6, 8	128.6, CH	7.41, t (7.5)		6, 8	128.6, CH	7.42, d (7.5)
	7	126.8, CH	7.24, t (7.5)		7	126.8, CH	7.24, d (7.5)
	NH		8.06, d (8.7)		NH		8.09, d (8.8)
Hmp	1	169.1, C		Hmp	1	169.1, C	
	2	75.6, CH	5.52, br. s		2	75.6, CH	5.54, d (1.5)
	3	36.4, CH	2.31, m		3	36.4, CH	2.32, m
	4	26.1, CH_2_	1.15, m		4	26.1, CH_2_	1.18, m
	5	11.6, CH_3_	0.70, t (7.3)		5	11.6, CH_3_	0.72, (7.4)
	3-Me	14.4, CH_3_	0.88, d (6.5)		3-Me	14.4, CH_3_	0.89, d (6.7)

^a–d^ These carbon signals are interchangeable.

## Data Availability

Not applicable.

## Supplementary Materials

The following supporting information can be downloaded at: https://www.mdpi.com/article/10.3390/md20100591/s1, Figures S1–S33: HRESIMS, ^1^H, ^13^C, enlarged ^13^C, DEPT135, HSQC, COSY, HMBC, and ROESY spectra of compounds **1** and **2**. Figure S34: Comparison of ^1^H NMR spectra of **1** and **3a** in pyridine-*d*_5_ at 600 MHz. Figure S35: HPLC chromatograms of the Marfey’s derivatives of **1** and **2**.
