# Supplementary material for: Phaeosphamides A and B, Cytotoxic Cyclodecadepsipeptides from the Mangrove-Derived Fungus Phaeosphaeriopsis sp. S296
Source: Mar Drugs. 2022 Sep 21;20(10):591. doi: 10.3390/md20100591 (PMC9604837; doi:10.3390/md20100591)
Supplement: Supplementary file 1 [file marinedrugs-20-00591-s001.zip › marinedrugs-1892090-supplementary.pdf]

Supporting Information

Phaeosphamides A and B, Cytotoxic Cyclodecadepsipeptides from the Mangrove-Derived Fungus *Phaeosphaeriopsis* sp. S296

Siwen Niu [1,†], Jianlin He [1,†], Shuhuan Huang [1], Shouyuan Wu [2], Ling Zeng [2], Juan Wang [1], Bihong Hong [1] and Ziming Chen [2,3,*]

[1] Technology Innovation Center for Exploitation of Marine Biological Resources, Third Institute of Oceanography, Ministry of Natural Resources, Xiamen 361005, China; niusiwen@tio.org.cn (S.N.); jlhe@tio.org.cn (J.H.); huangsh2268@163.com (S.H.); wangjuan@tio.org.cn (J.W.); bhhong@tio.org.cn (B.H.)

[2] School of Chemistry and Chemical Engineering, Lingnan Normal University, Zhanjiang 524048, China; wushouyuan2012@163.com (S.W.); karine126@126.com (L.Z.)

[3] Mangrove Institute, Lingnan Normal University, Zhanjiang 524048, China

* Correspondence: chenzm818@163.com; Tel.: +86-075-9318-3245

† The authors contributed equally to this work.

Contents **Pages**

Figure S1. HRESIMS spectrum of **1**. S4

Figure S2. ^{1}H NMR spectrum of **1** in pyridine-d_5 (400 MHz). S5

Figure S3. ^{13}C NMR spectrum of **1** in pyridine-d_5 (100 MHz). S6

Figure S4. Enlarged ^{13}C NMR spectrum of **1** in pyridine-d_5 (100 MHz). S7

Figure S5. Enlarged ^{13}C NMR spectrum of **1** in pyridine-d_5 (100 MHz). S8

Figure S6. Enlarged ^{13}C NMR spectrum of **1** in pyridine-d_5 (100 MHz). S9

Figure S7. DEPT135 NMR spectrum of **1** in pyridine-d_5 (100 MHz). S10

Figure S8. Enlarged DEPT135 NMR spectrum of **1** in pyridine-d_5. S11

Figure S9. HSQC spectrum of **1** in pyridine-d_5. S12

Figure S10. ^{1}H-^{1}H COSY spectrum of **1** in pyridine-d_5. S13

Figure S11. HMBC spectrum of **1** in pyridine-d_5. S14

Figure S12. Enlarged HMBC spectrum of **1** in pyridine-d_5. S15

Figure S13. Enlarged HMBC spectrum of **1** in pyridine-d_5. S16

Figure S14. Enlarged HMBC spectrum of **1** in pyridine-d_5. S17

Figure S15. ROESY spectrum of **1** in pyridine-d_5. S18

Figure S16. Enlarged ROESY spectrum of **1** in pyridine-d_5. S19

Figure S17. HRESIMS spectrum of **2**. S20

Figure S18. ^{1}H NMR spectrum of **2** in pyridine-d_5 (400 MHz). S21

Figure S19. ^{13}C NMR spectrum of **2** in pyridine-d_5 (100 MHz). S22

Figure S20. Enlarged ^{13}C NMR spectrum of **2** in pyridine-d_5. S23

Figure S21. Enlarged ^{13}C NMR spectrum of **2** in pyridine-d_5. S24

Figure S22. Enlarged ^{13}C NMR spectrum of **2** in pyridine-d_5. S25

Figure S23. DEPT135 NMR spectrum of **2** in pyridine-d_5 (100 MHz). S26

Figure S24. Enlarged DEPT135 NMR spectrum of **2** in pyridine-d_5. S27

Figure S25. Enlarged DEPT135 NMR spectrum of **2** in pyridine-d_5. S28

Figure S26. HSQC spectrum of **2** in pyridine-d_5. S29

Figure S27. ^{1}H-^{1}H COSY spectrum of **2** in pyridine-d_5. S30

Figure S28. HMBC spectrum of **2** in pyridine-d_5. S31

Figure S29. Enlarged HMBC spectrum of **2** in pyridine-d_5. S32

Figure S30. Enlarged HMBC spectrum of **2** in pyridine-d_5. S33

Figure S31. Enlarged HMBC spectrum of **2** in pyridine-d_5. S34

Figure S32. ROESY spectrum of **2** in pyridine-d_5. S35

Figure S33. Enlarged ROESY spectrum of **2** in pyridine-d_5. S36

Figure S34. Comparision of ^{1}H NMR spectra of **1** and **3a** in pyridine-d_5 at 600 MHz. S37

Figure S35. HPLC chromatograms of the Marfey's derivatives of **2** and **1**. S38

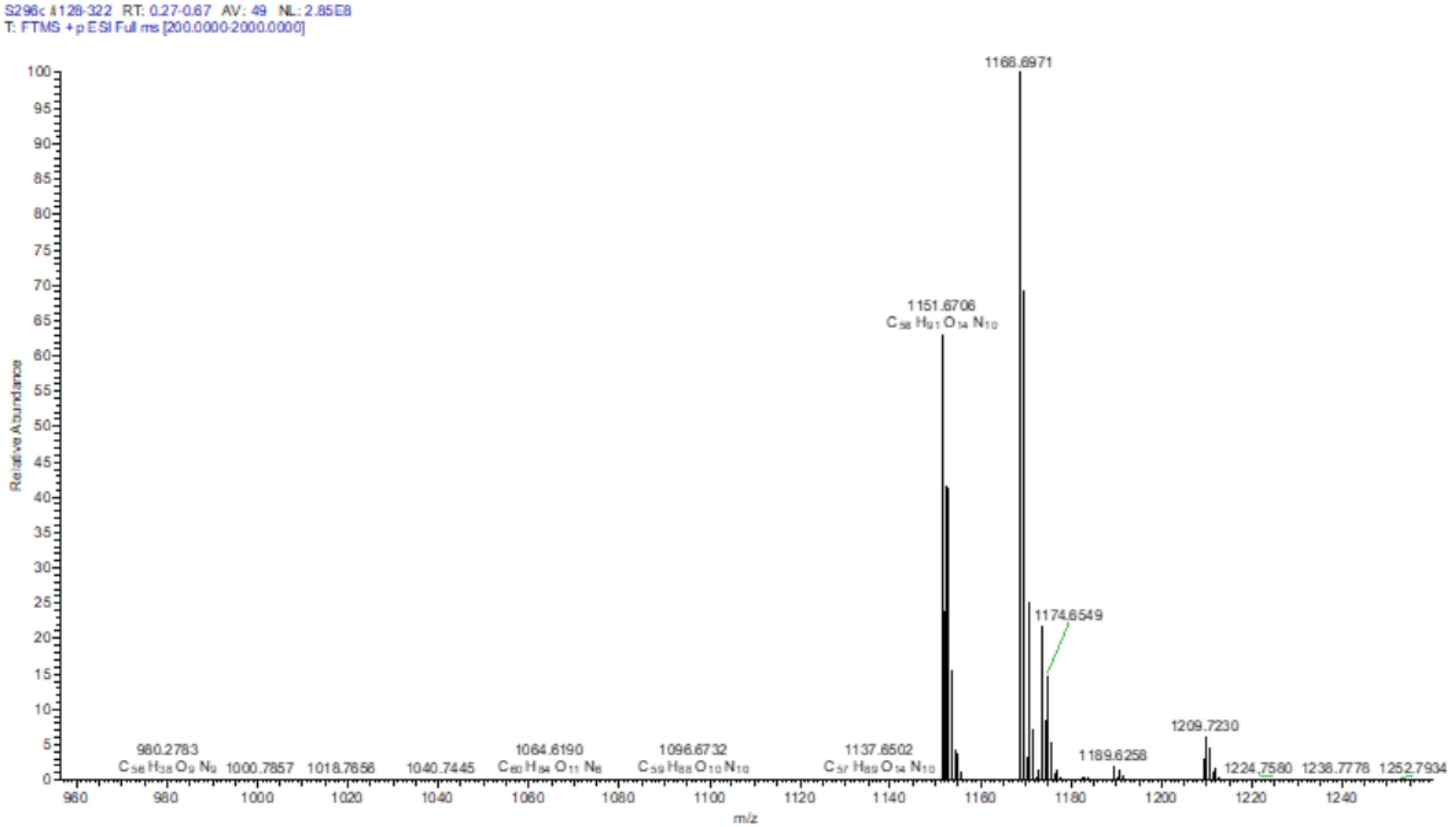

Figure S1. HRESIMS spectrum of **1**.

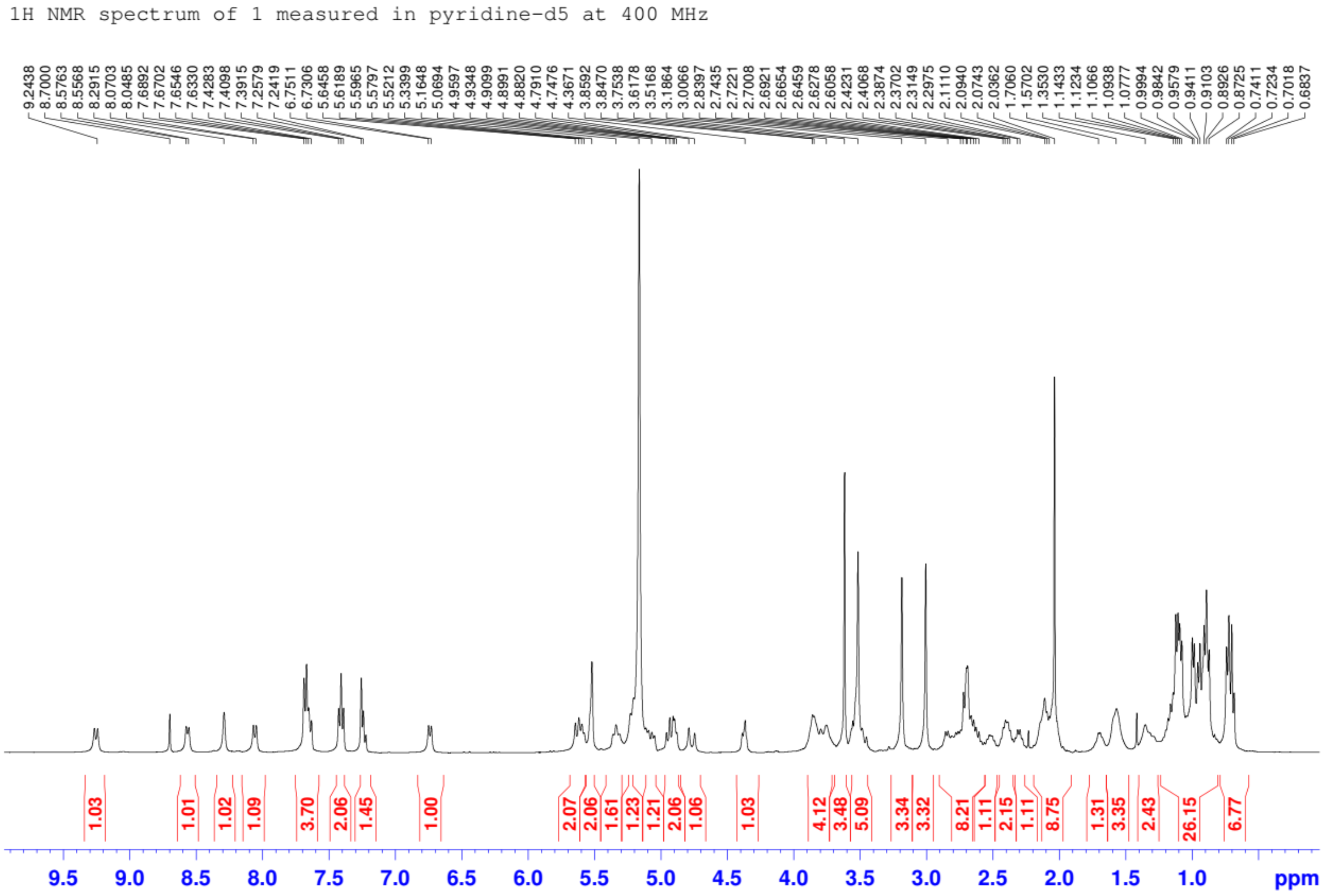

Figure S2. ^{1}H NMR spectrum of **1** in pyridine-d_5 (400 MHz).

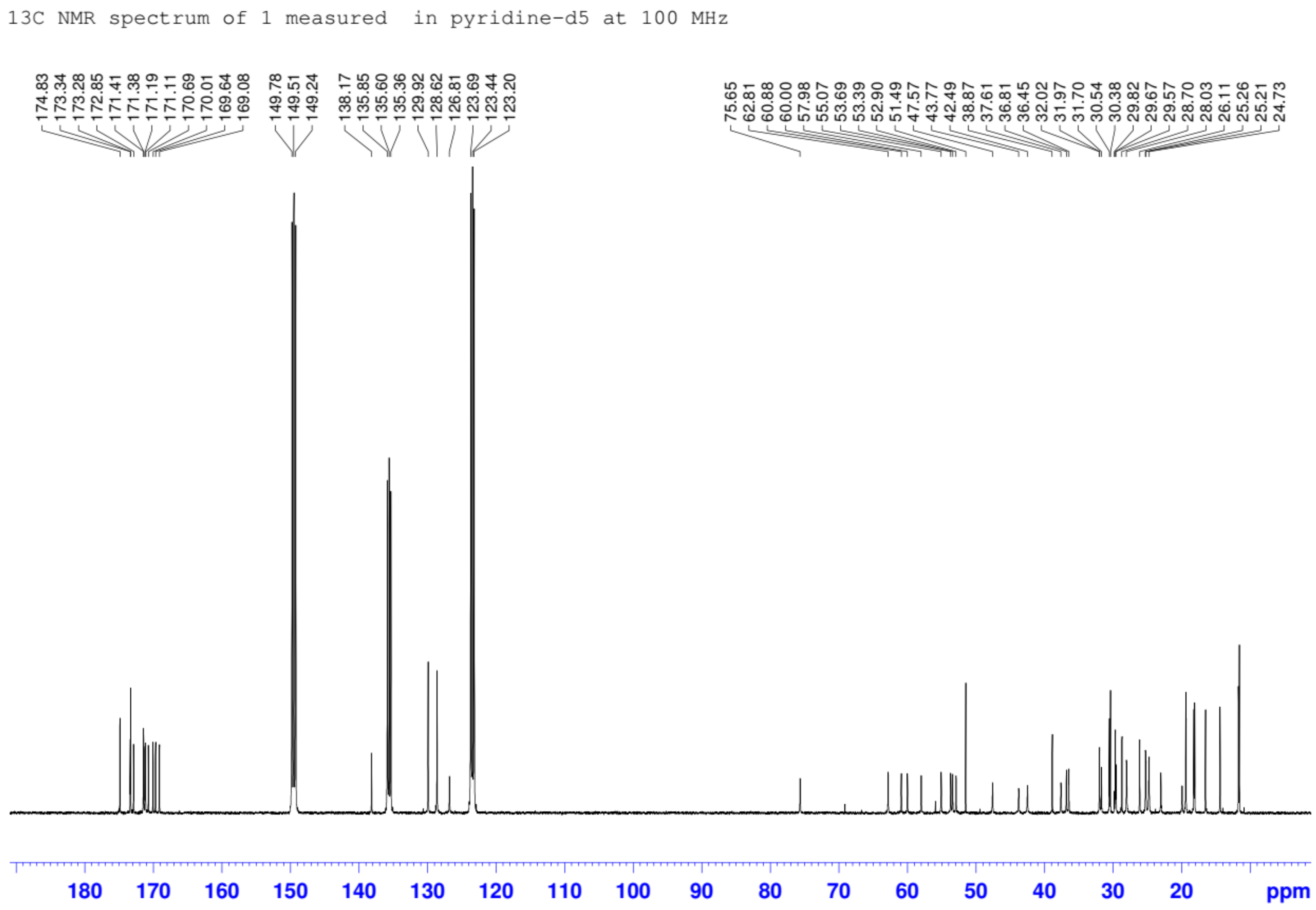

Figure S3. ^{13}C NMR spectrum of **1** in pyridine-d_5 (100 MHz).

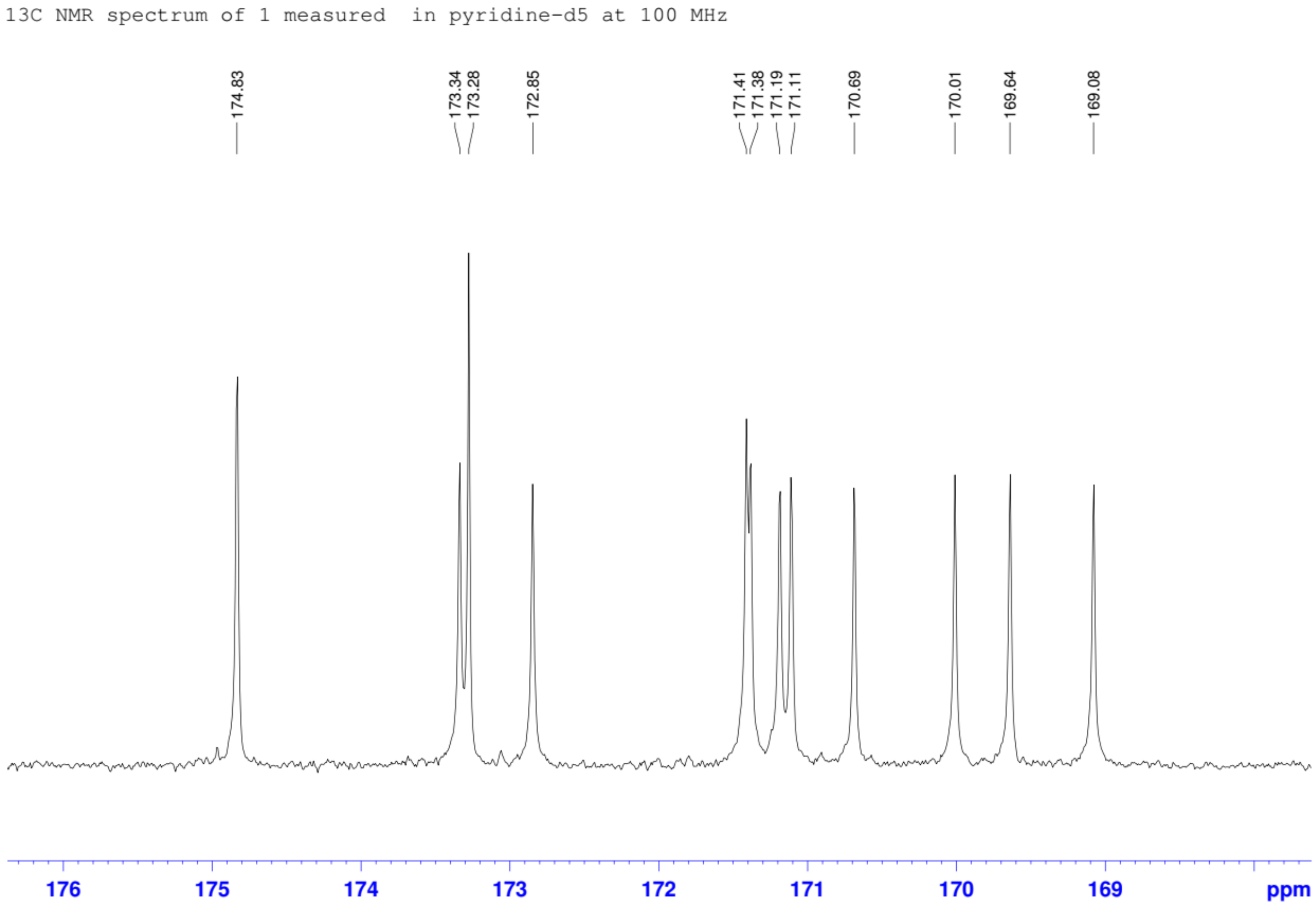

Figure S4. Enlarged ^{13}C NMR spectrum of **1** in pyridine-d_5 (100 MHz).

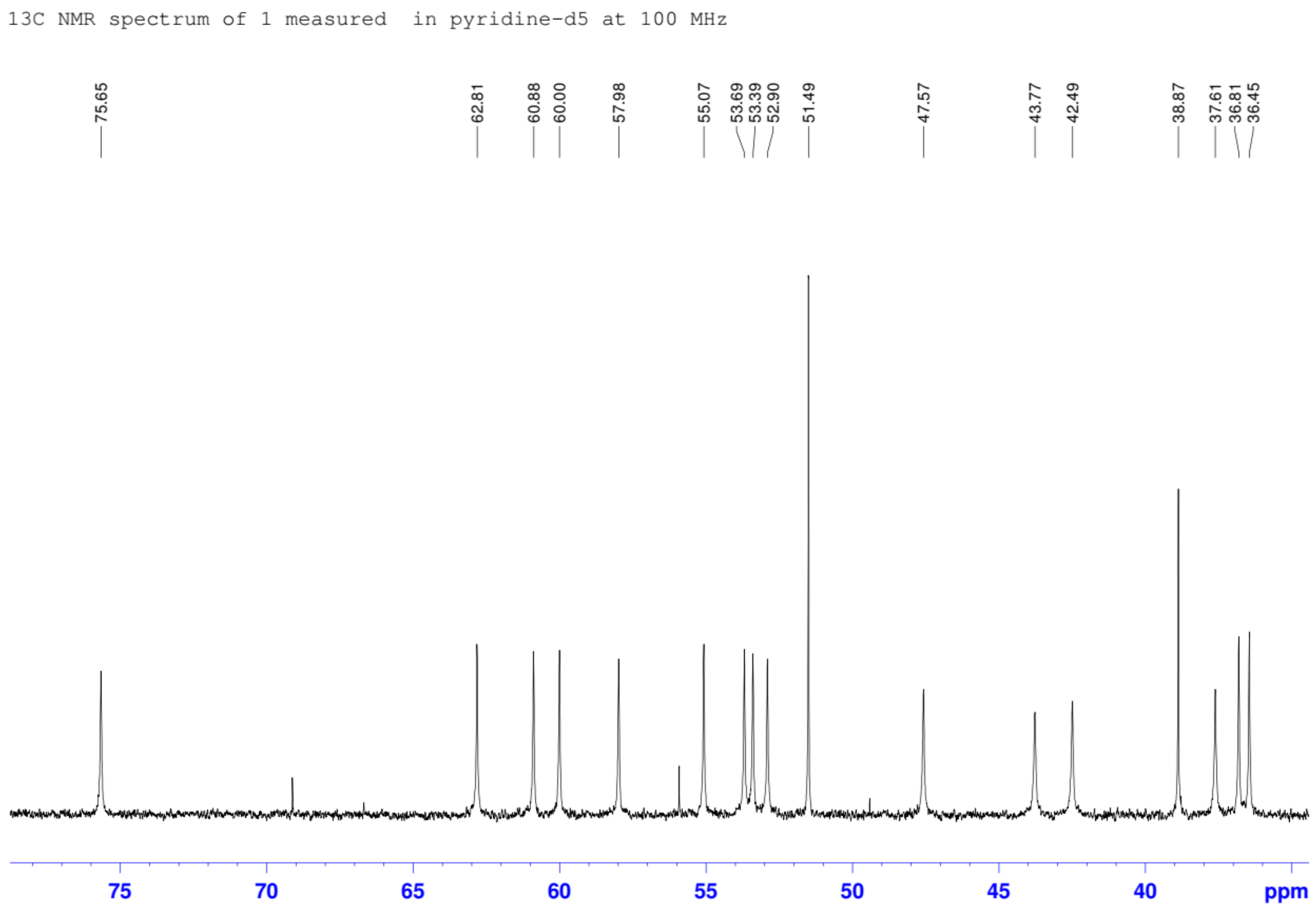

Figure S5. Enlarged ^{13}C NMR spectrum of **1** in pyridine-d_5 (100 MHz).

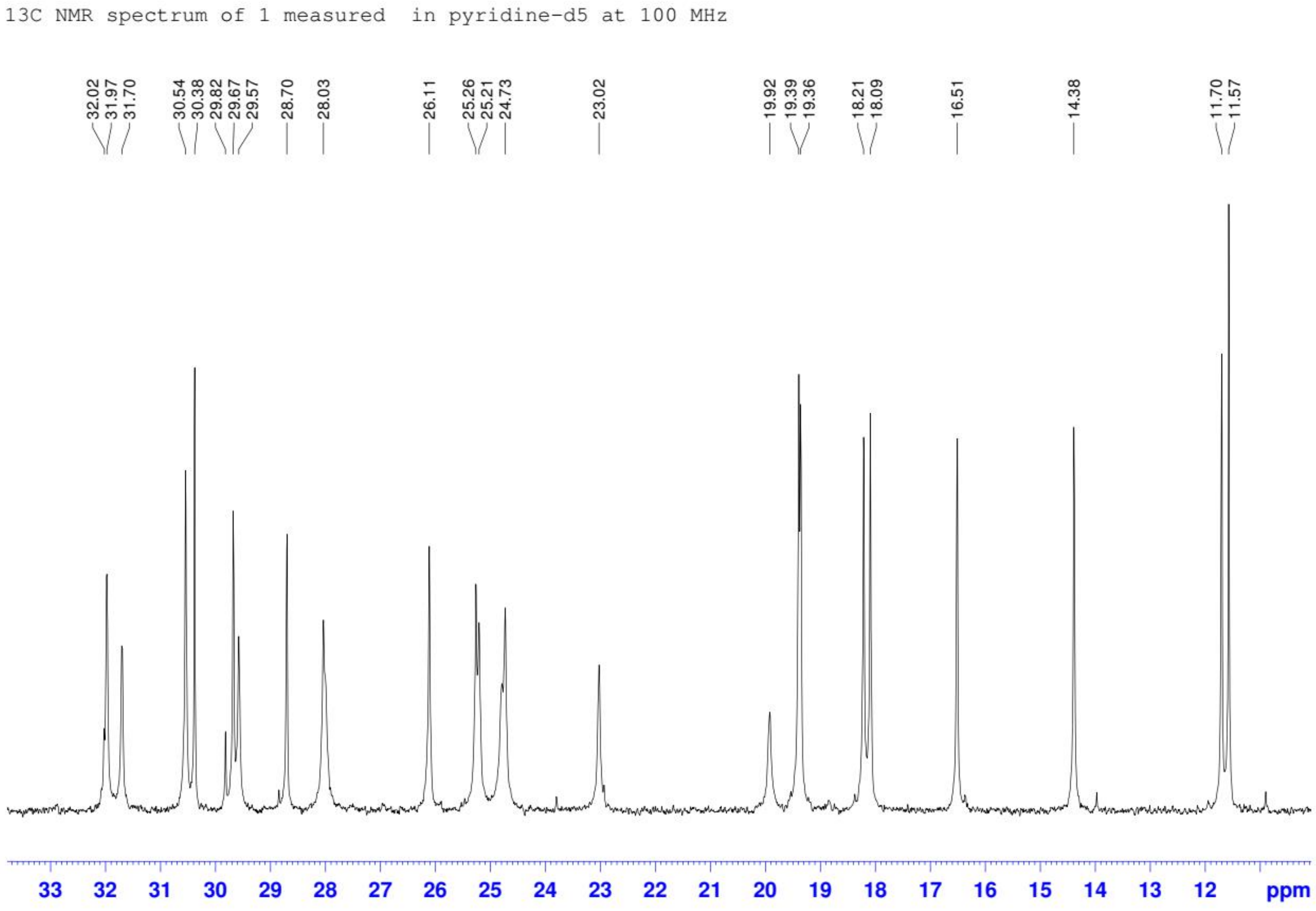

Figure S6. Enlarged ^{13}C NMR spectrum of **1** in pyridine-d_5 (100 MHz).

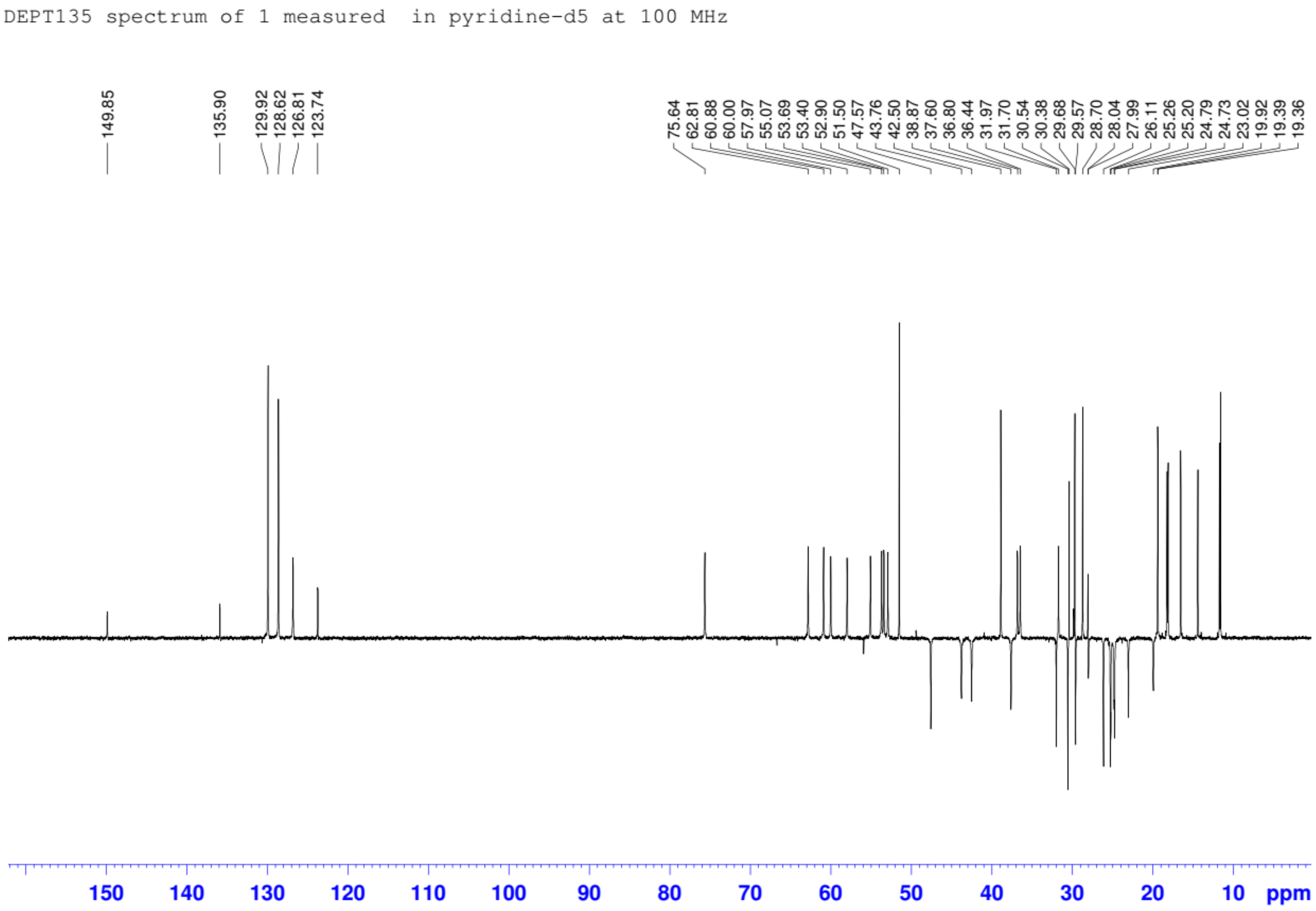

Figure S7. DEPT135 NMR spectrum of **1** in pyridine-d_5 (100 MHz).

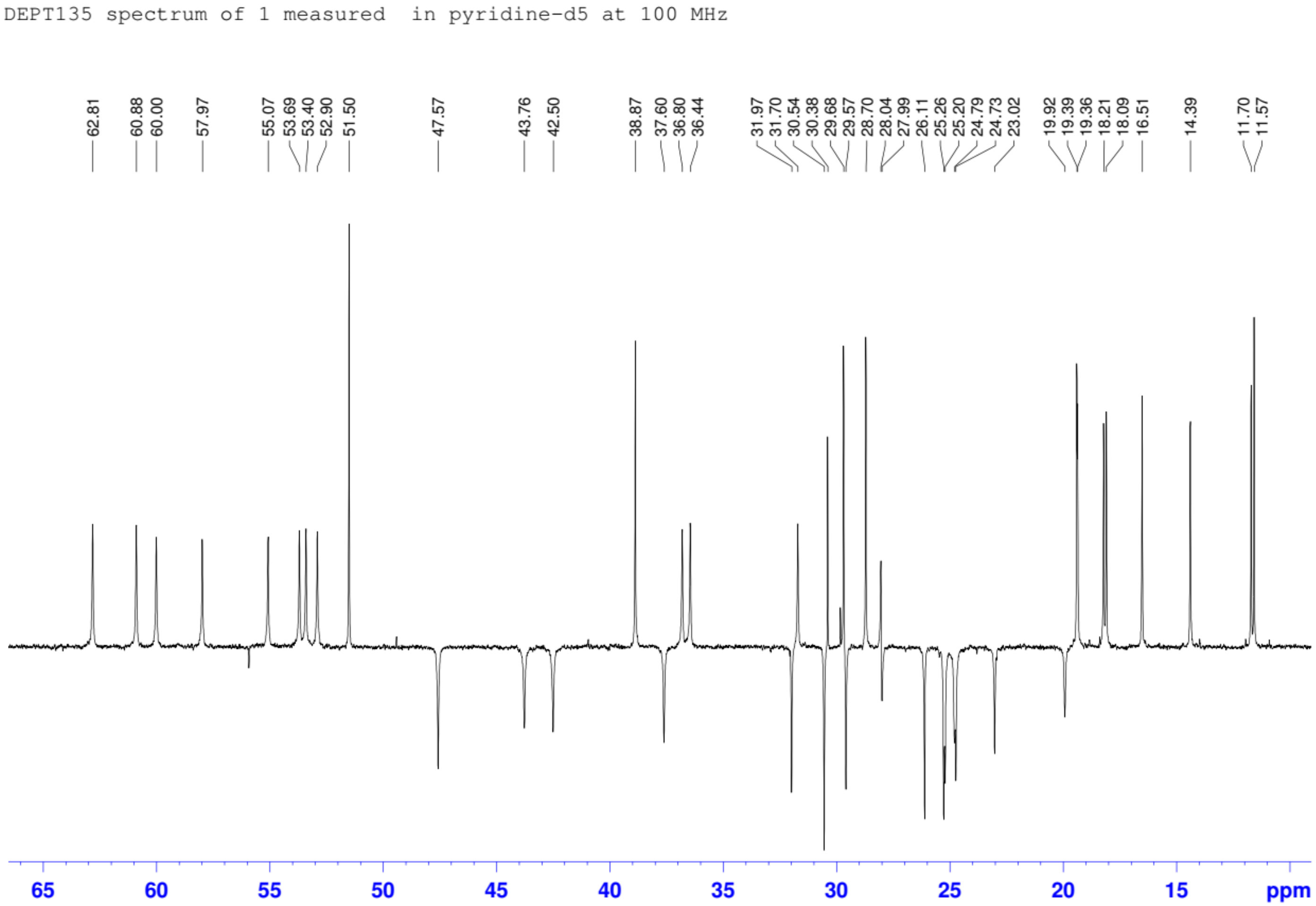

Figure S8. Enlarged DEPT135 NMR spectrum of **1** in pyridine-d_5 (100 MHz).

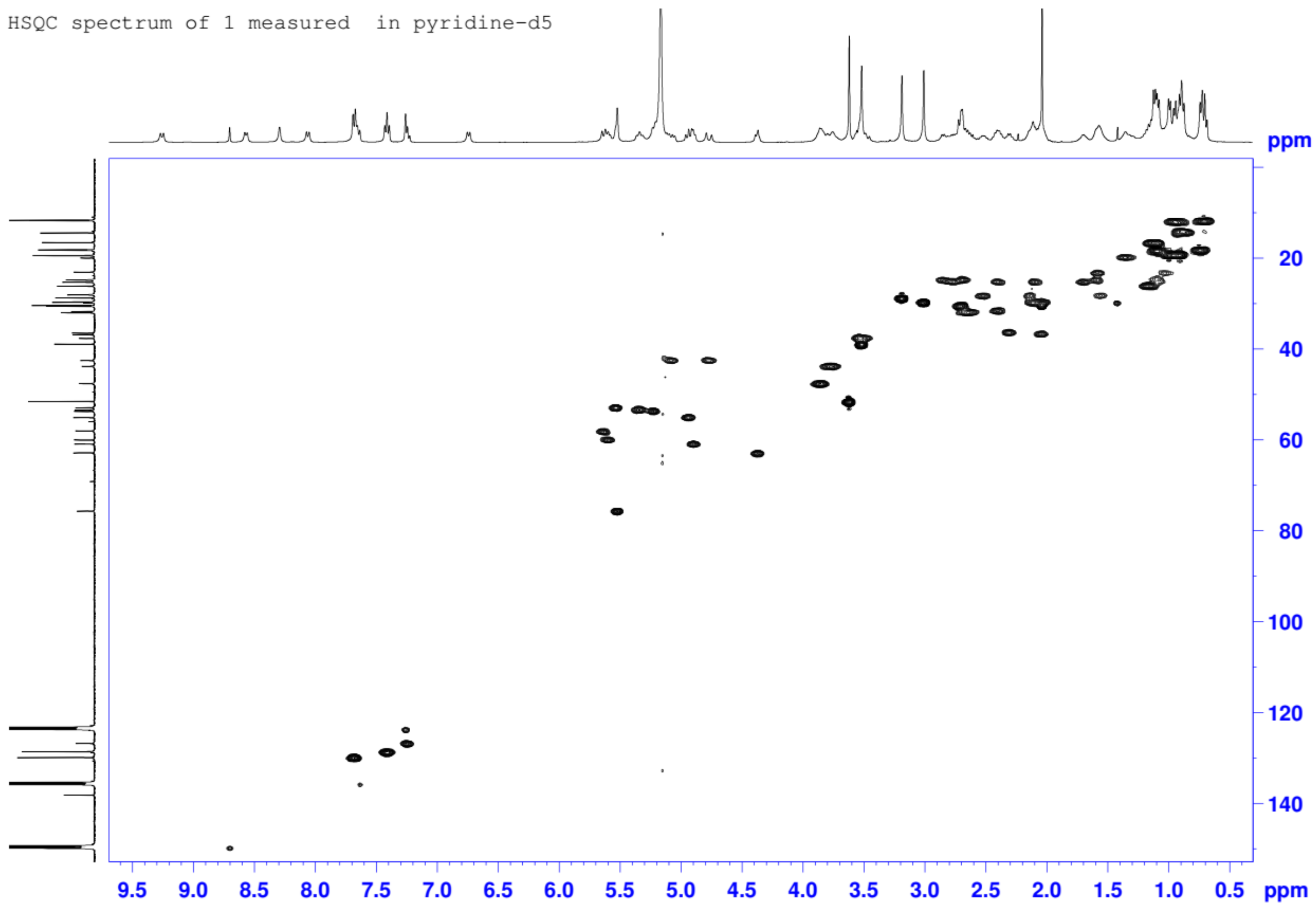

Figure S9. HSQC spectrum of **1** in pyridine-d_5.

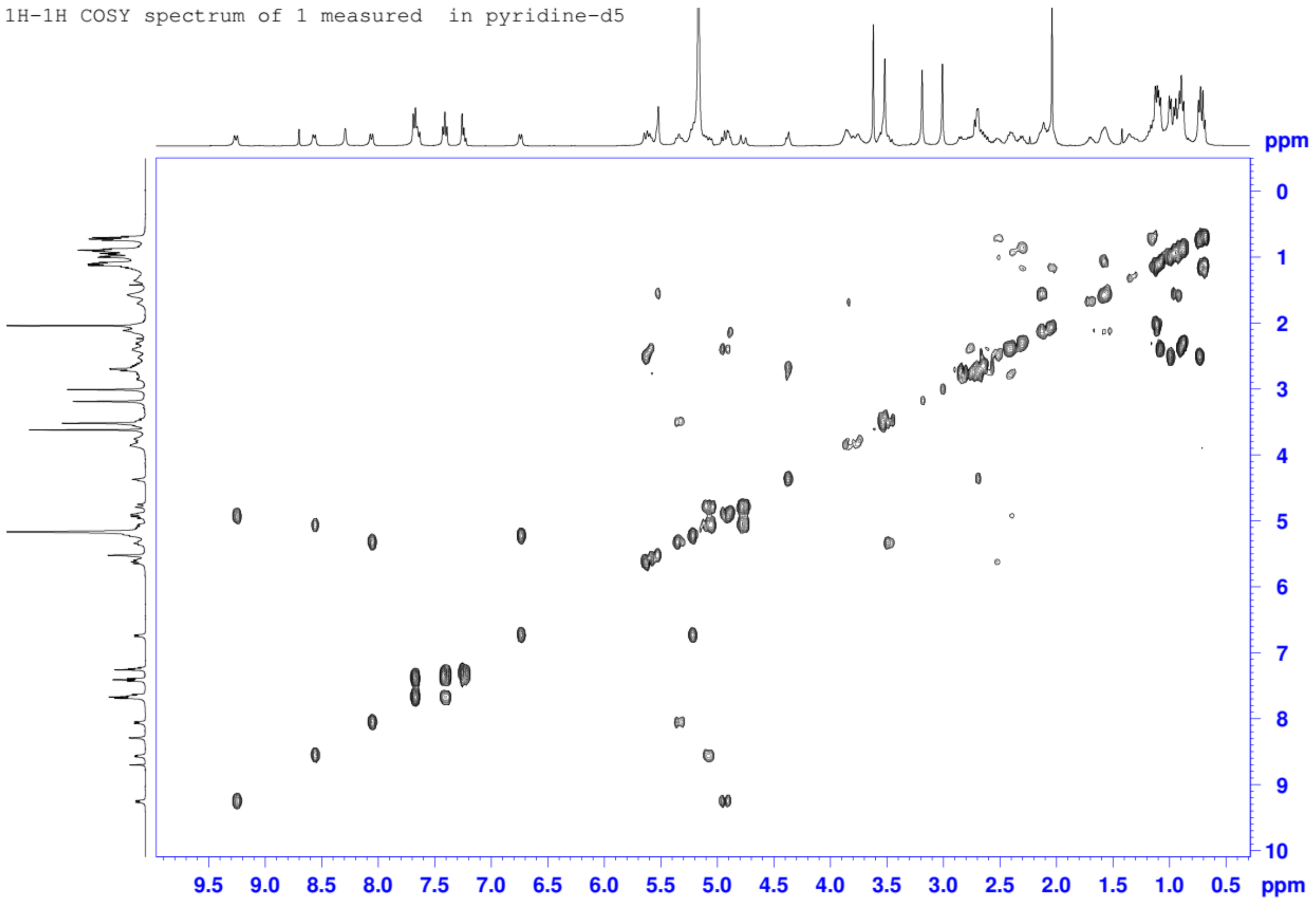

Figure S10. ^{1}H-^{1}H COSY spectrum of **1** in pyridine-d_5.

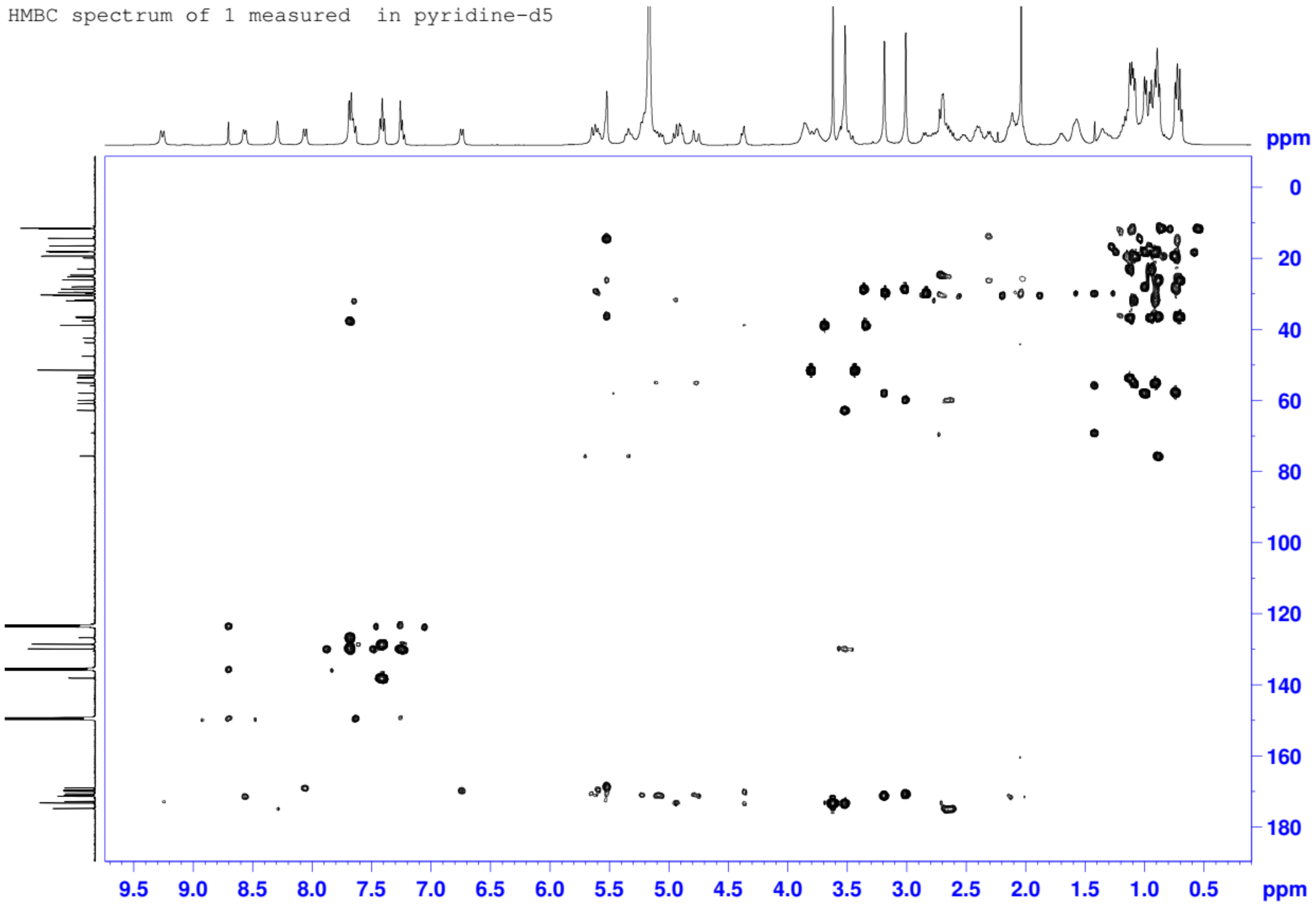

Figure S11. HMBC spectrum of **1** in pyridine-d_5.

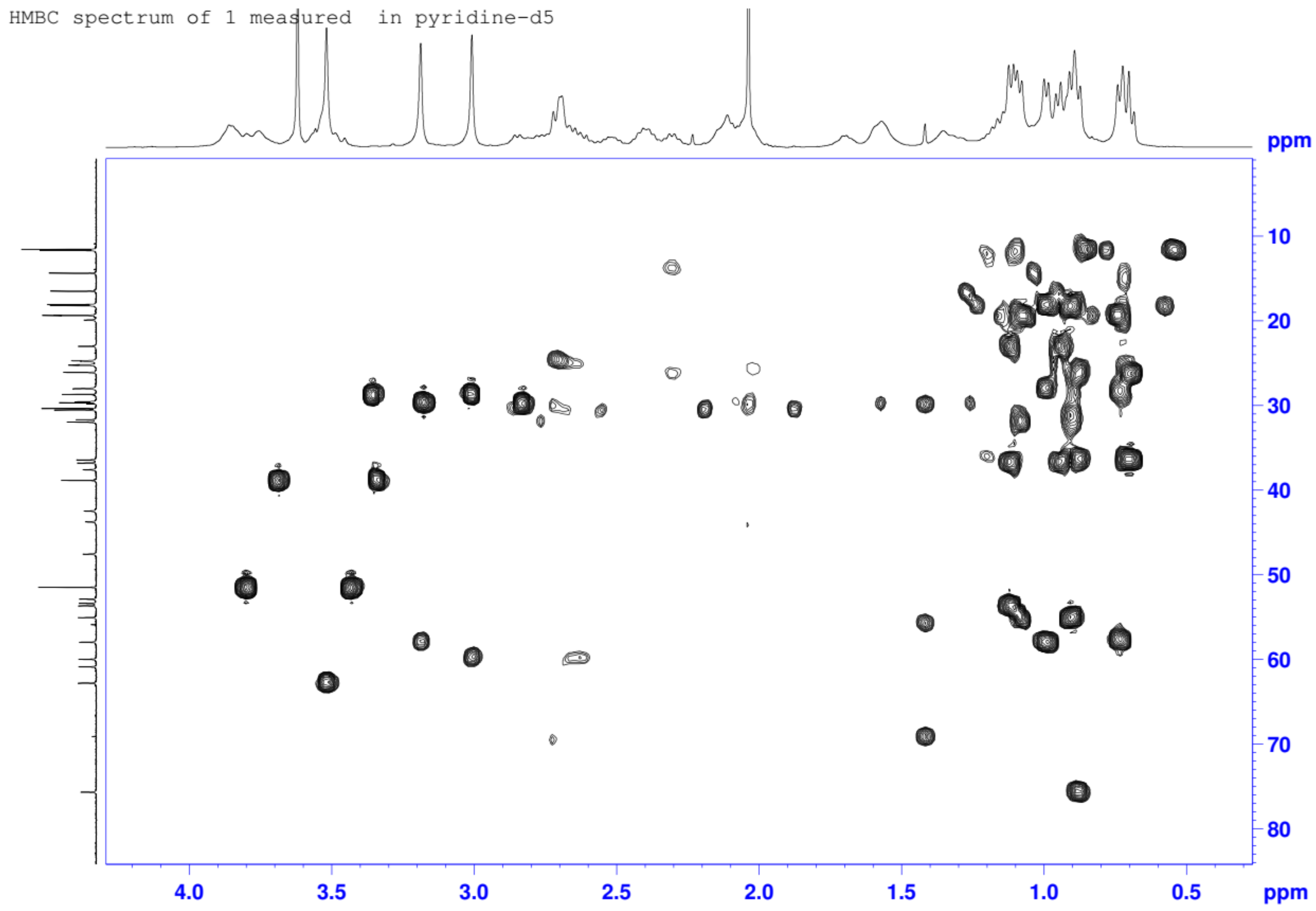

Figure S12. Enlarged HMBC spectrum of **1** in pyridine-d_5.

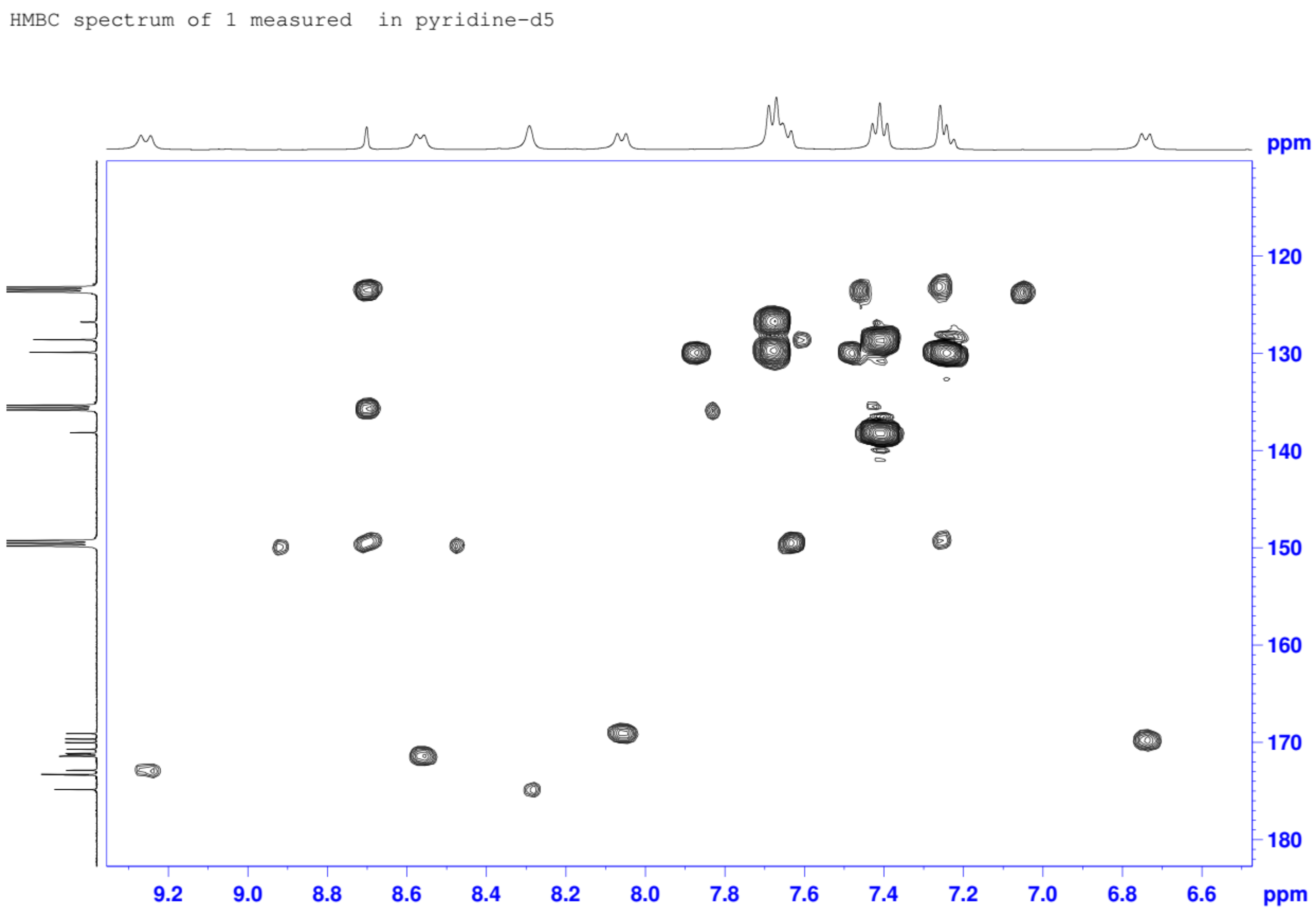

Figure S13. Enlarged HMBC spectrum of **1** in pyridine-d_5.

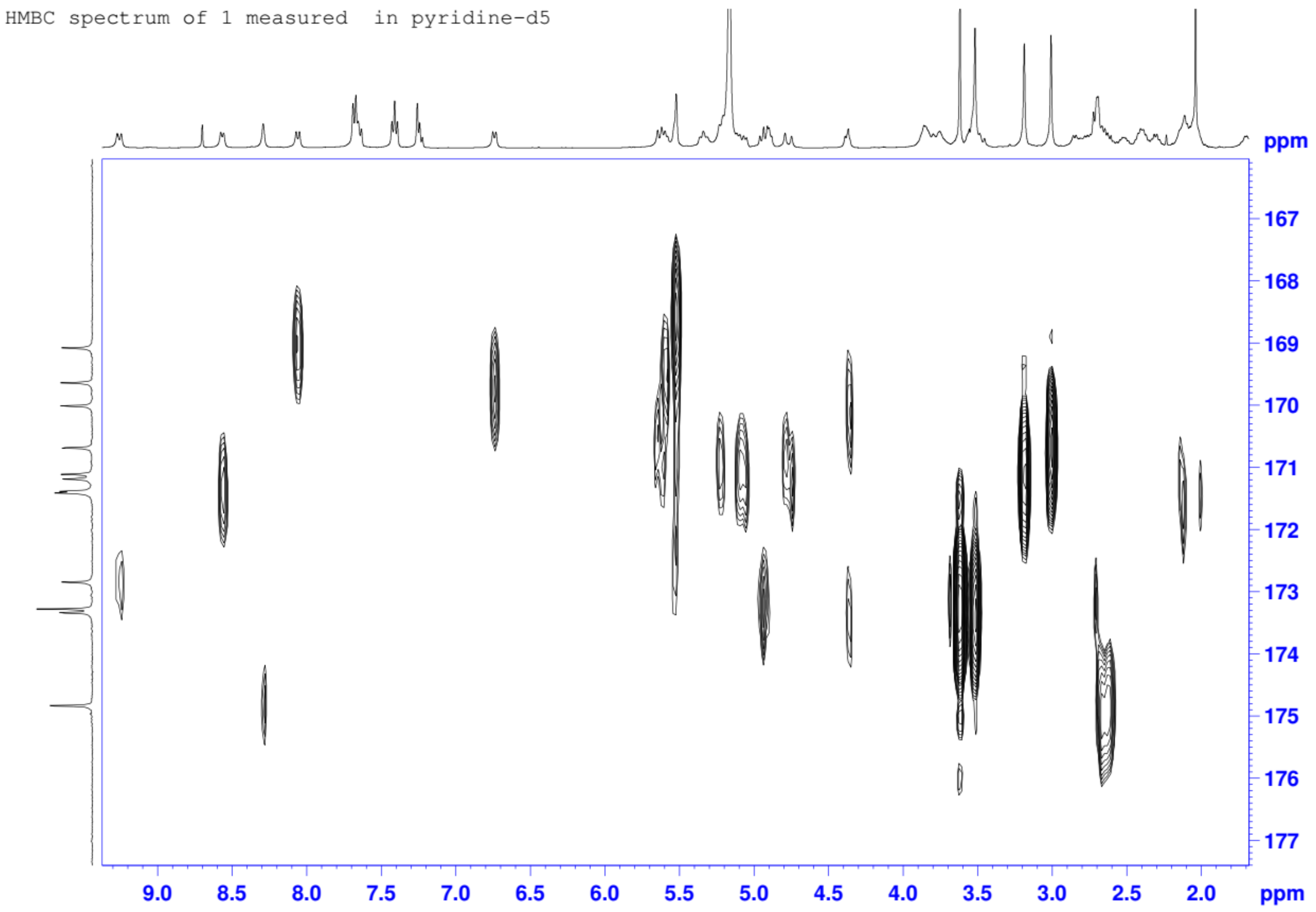

Figure S14. Enlarged HMBC spectrum of **1** in pyridine-d_5.

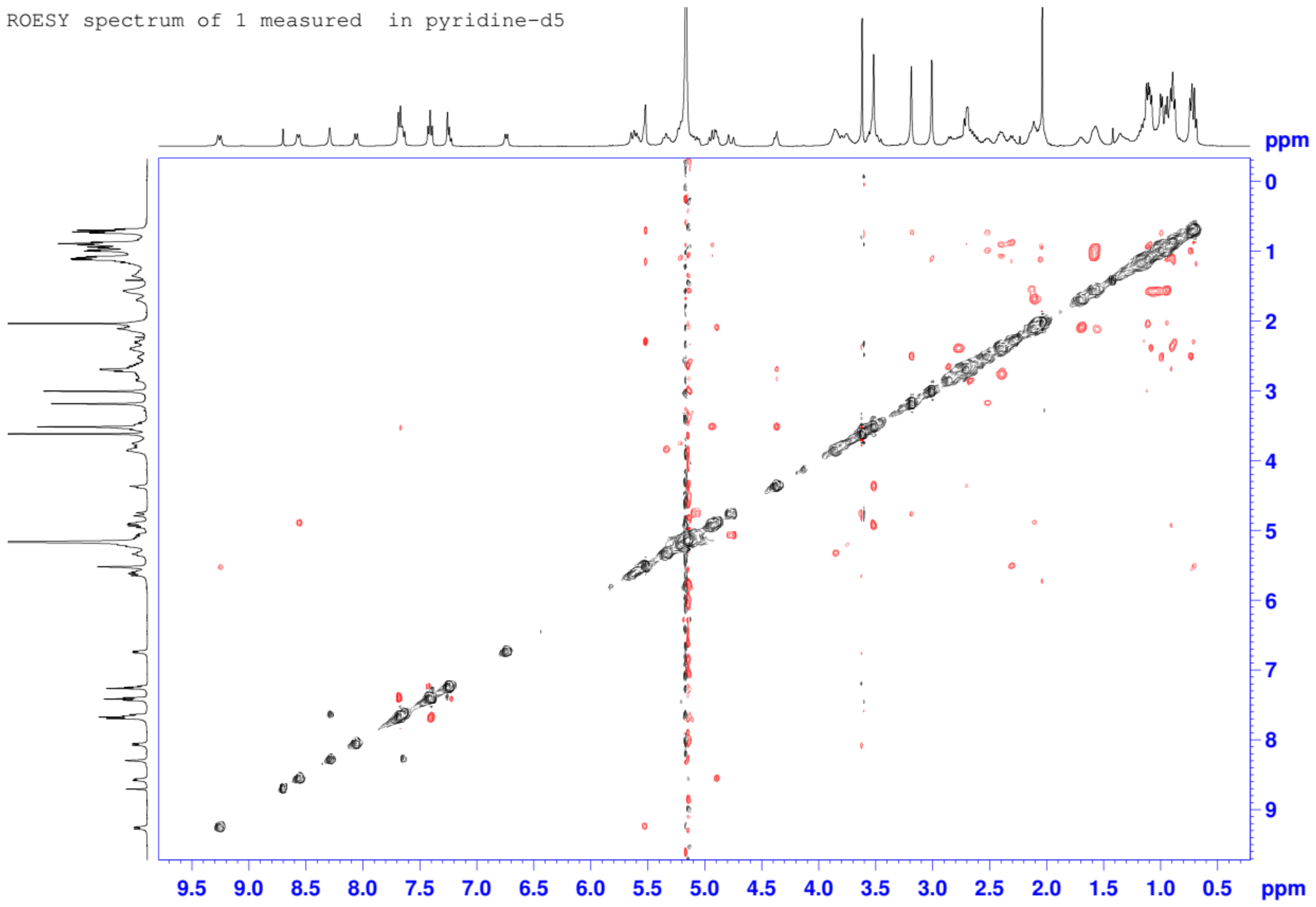

Figure S15. ROESY spectrum of **1** in pyridine-d_5.

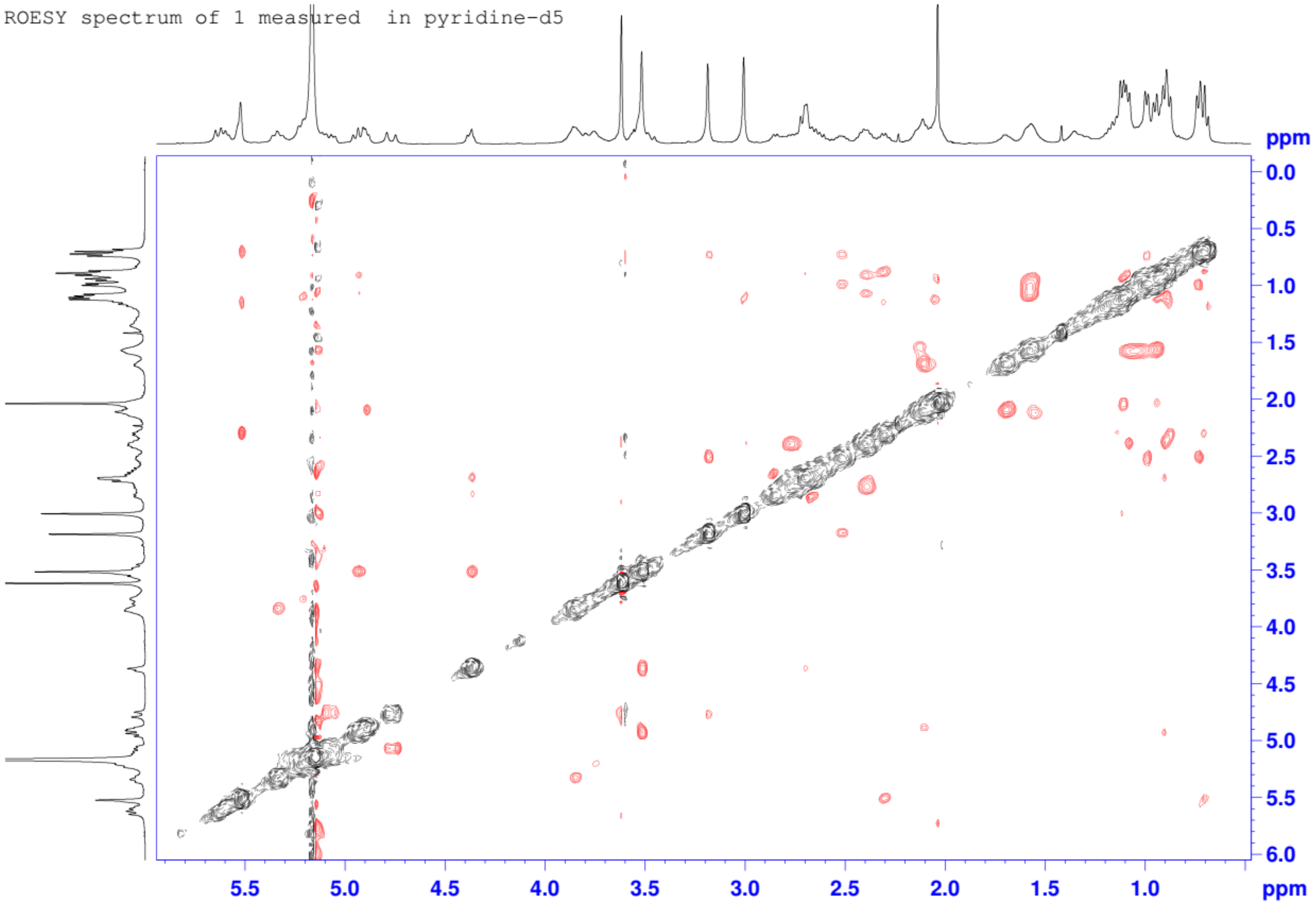

Figure S16. Enlarged ROESY spectrum of **1** in pyridine-d_5.

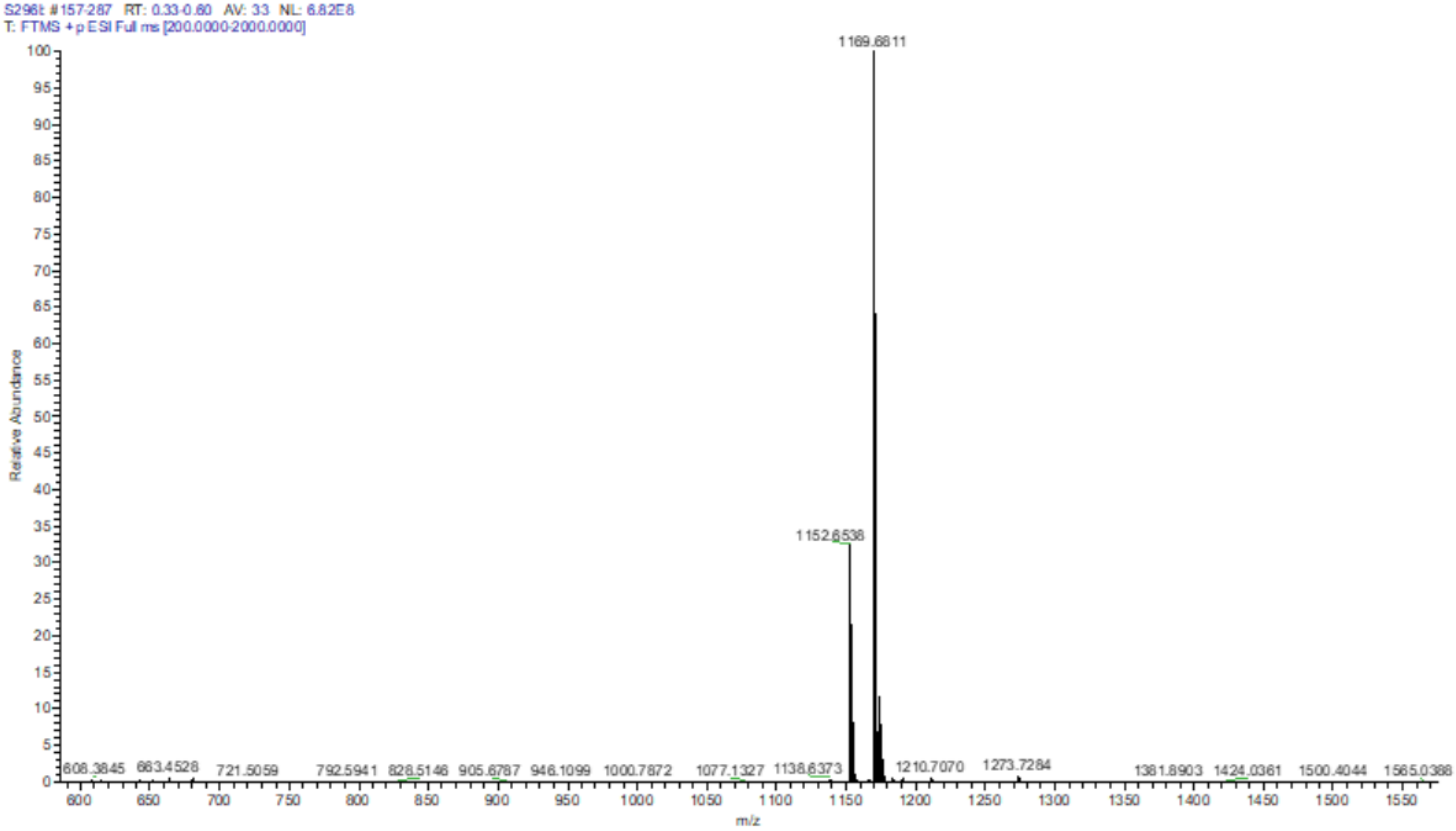

Figure S17. HRESIMS spectrum of **2**.

1H NMR spectrum of 2 measured in pyridine-d5 at 400 MHz

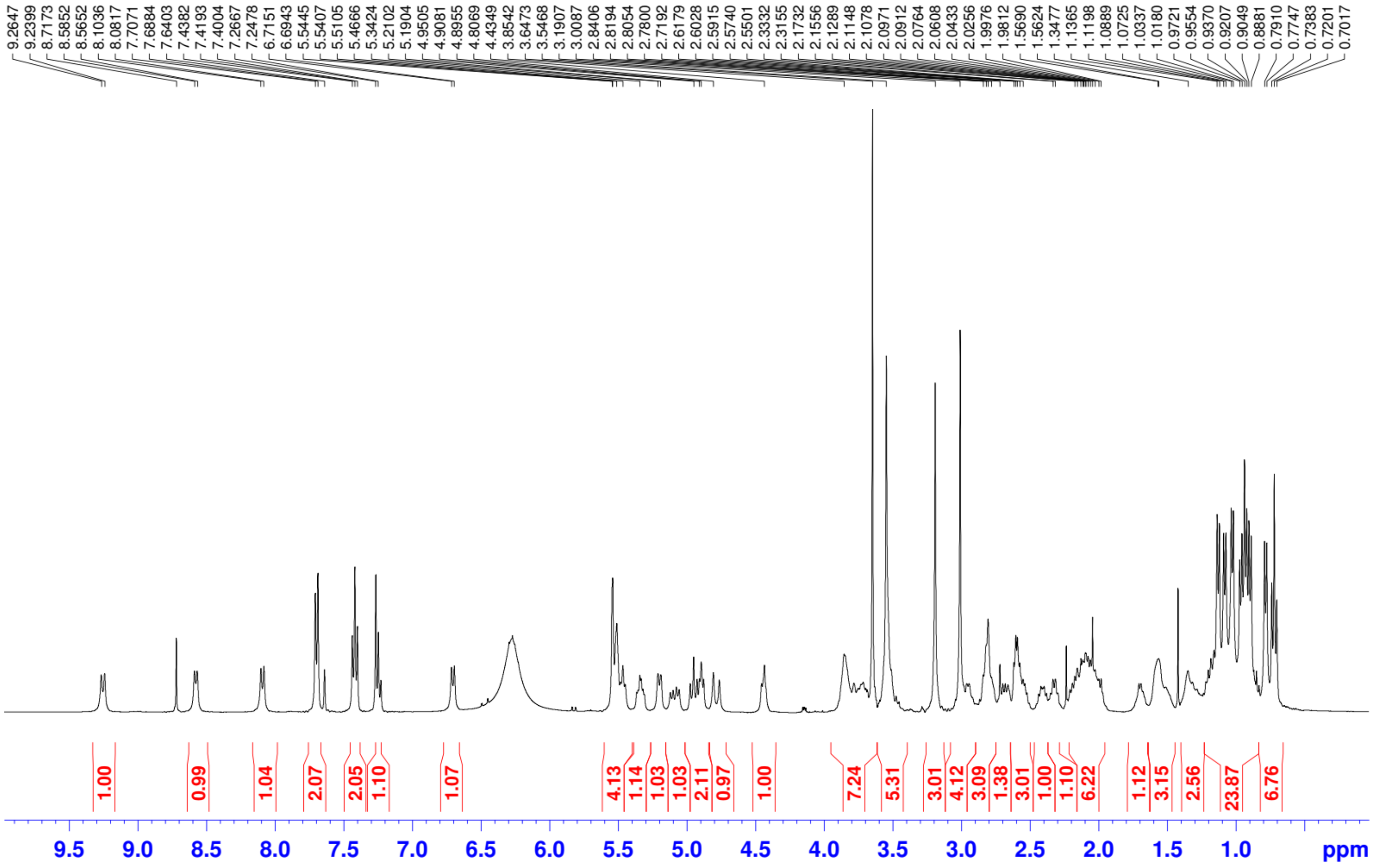

Figure S18. ^{1}H NMR spectrum of **2** in pyridine-d_5 (400 MHz).

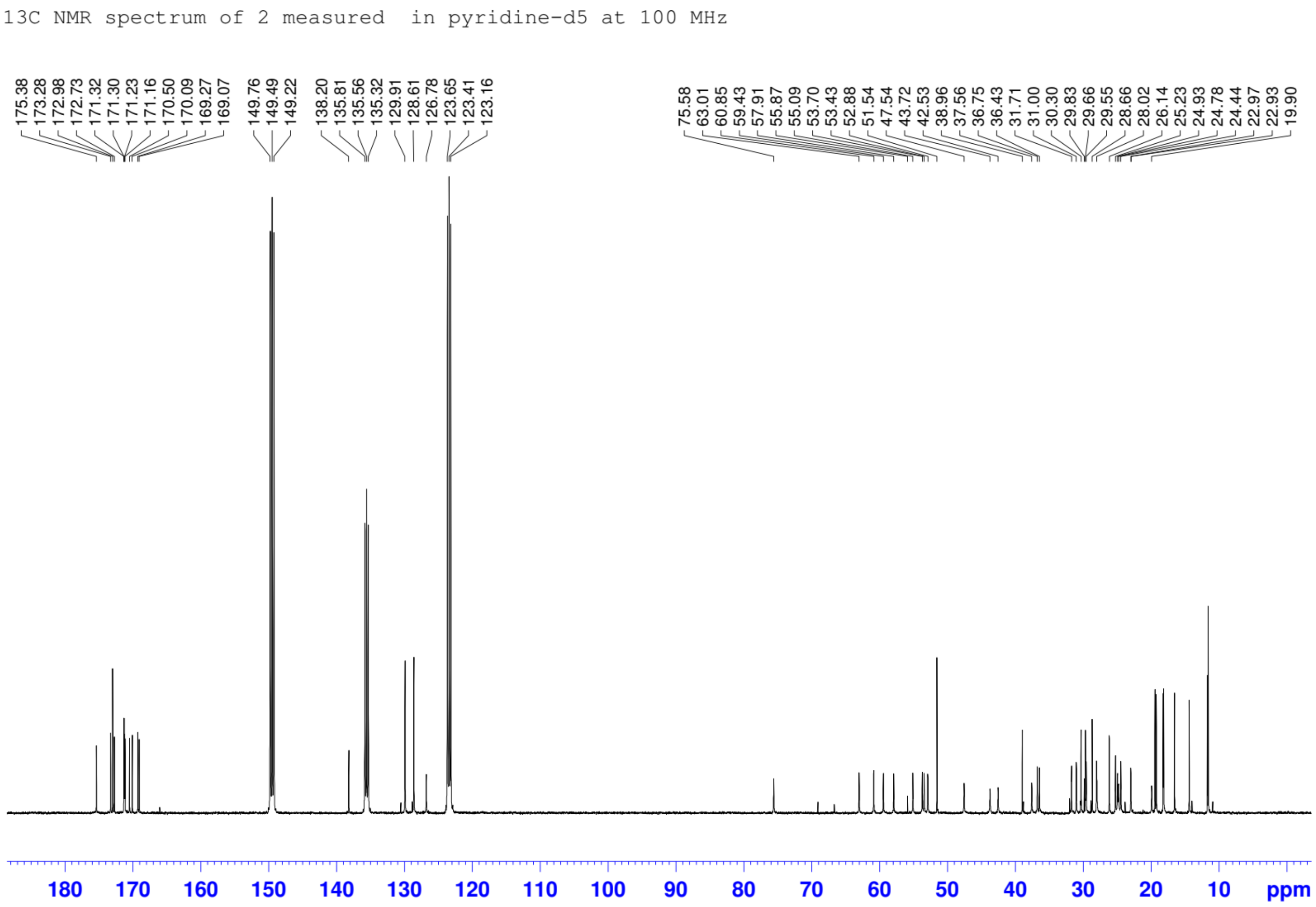

Figure S19. ^{13}C NMR spectrum of **2** in pyridine-d_5 (100 MHz).

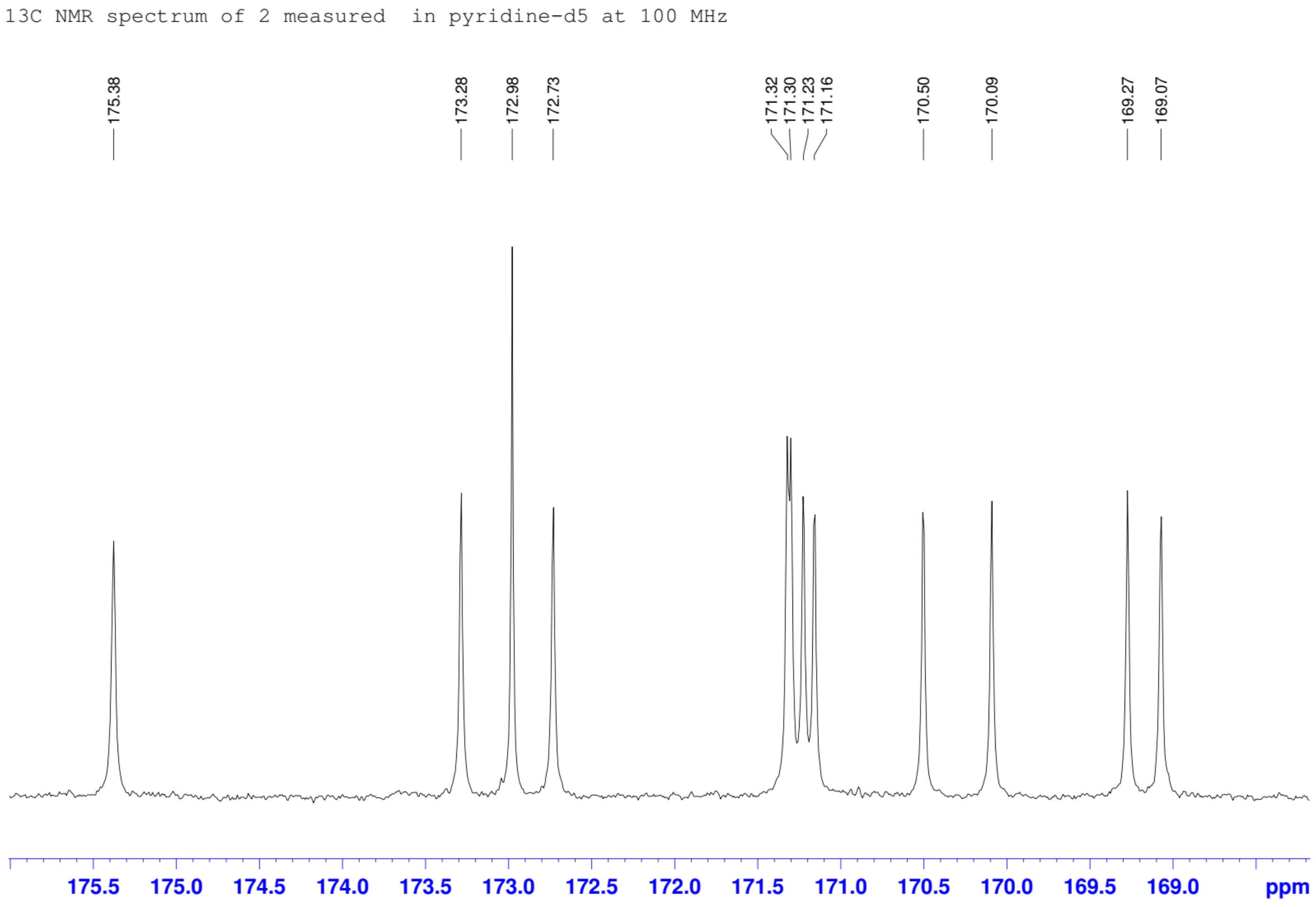

Figure S20. Enlarged ^{13}C NMR spectrum of **2** in pyridine-d_5 (100 MHz).

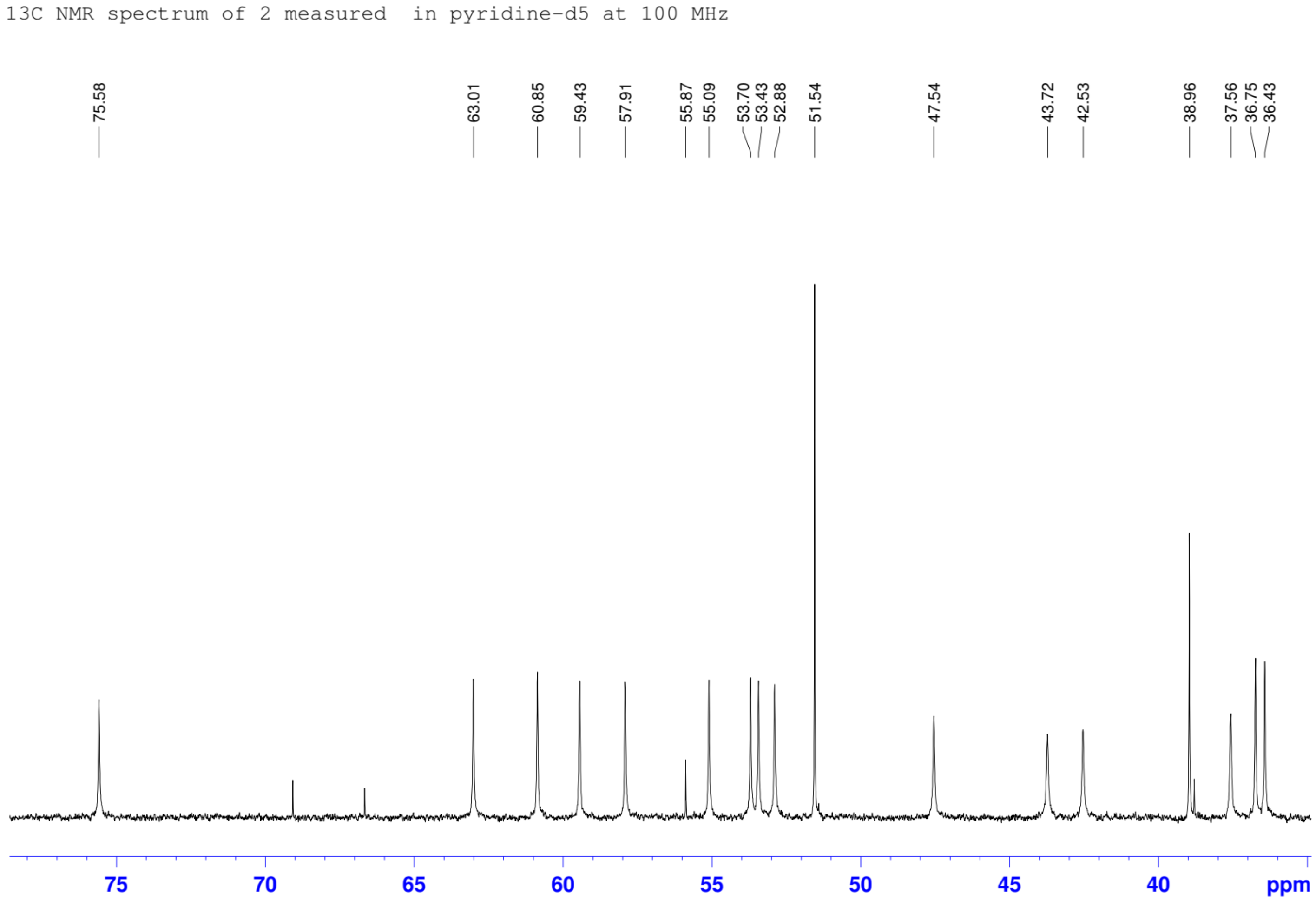

Figure S21. Enlarged ^{13}C NMR spectrum of **2** in pyridine-d_5 (100 MHz).

13C NMR spectrum of 2 measured in pyridine-d5 at 100 MHz

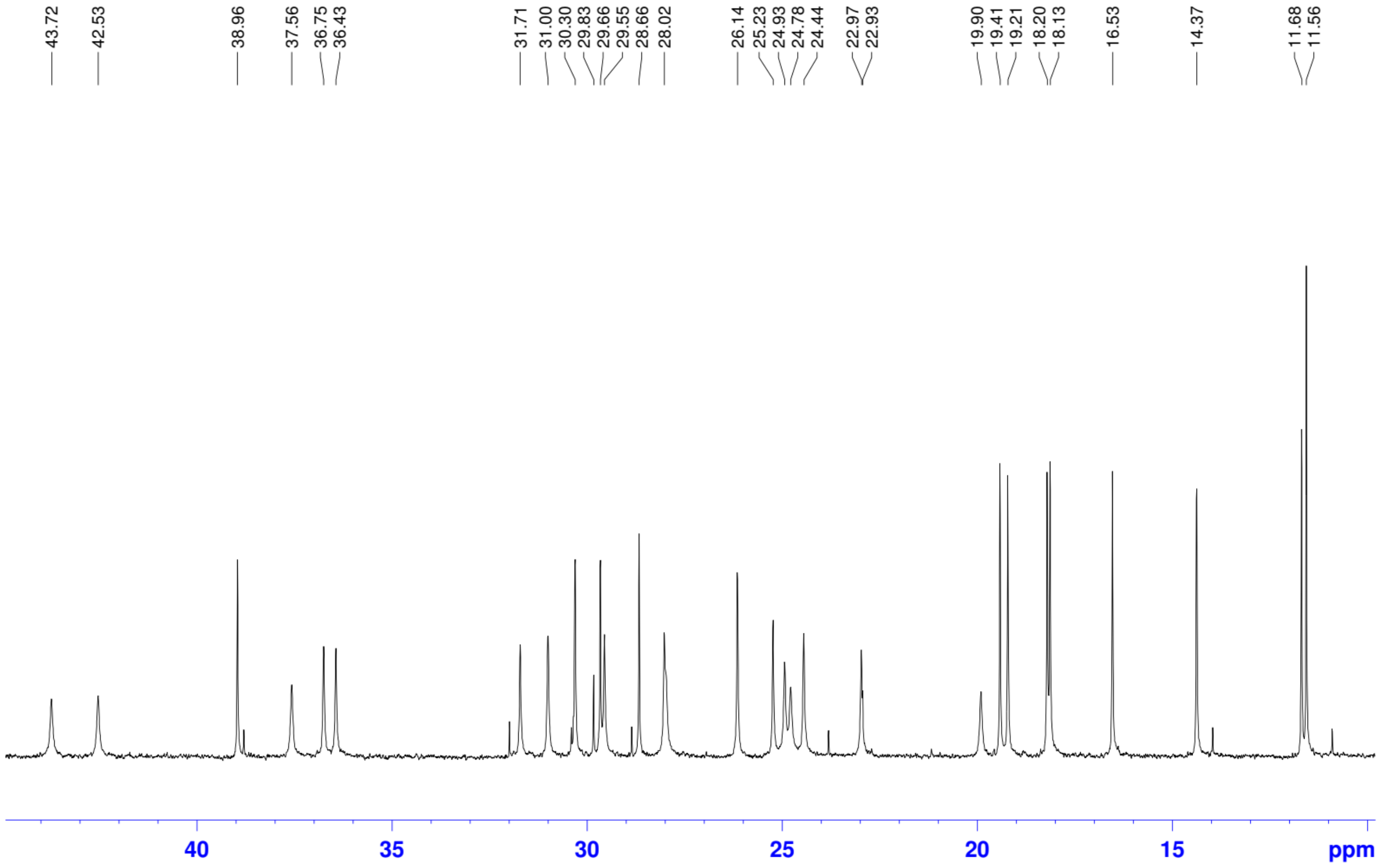

Figure S22. Enlarged ^{13}C NMR spectrum of **2** in pyridine-d_5 (100 MHz).

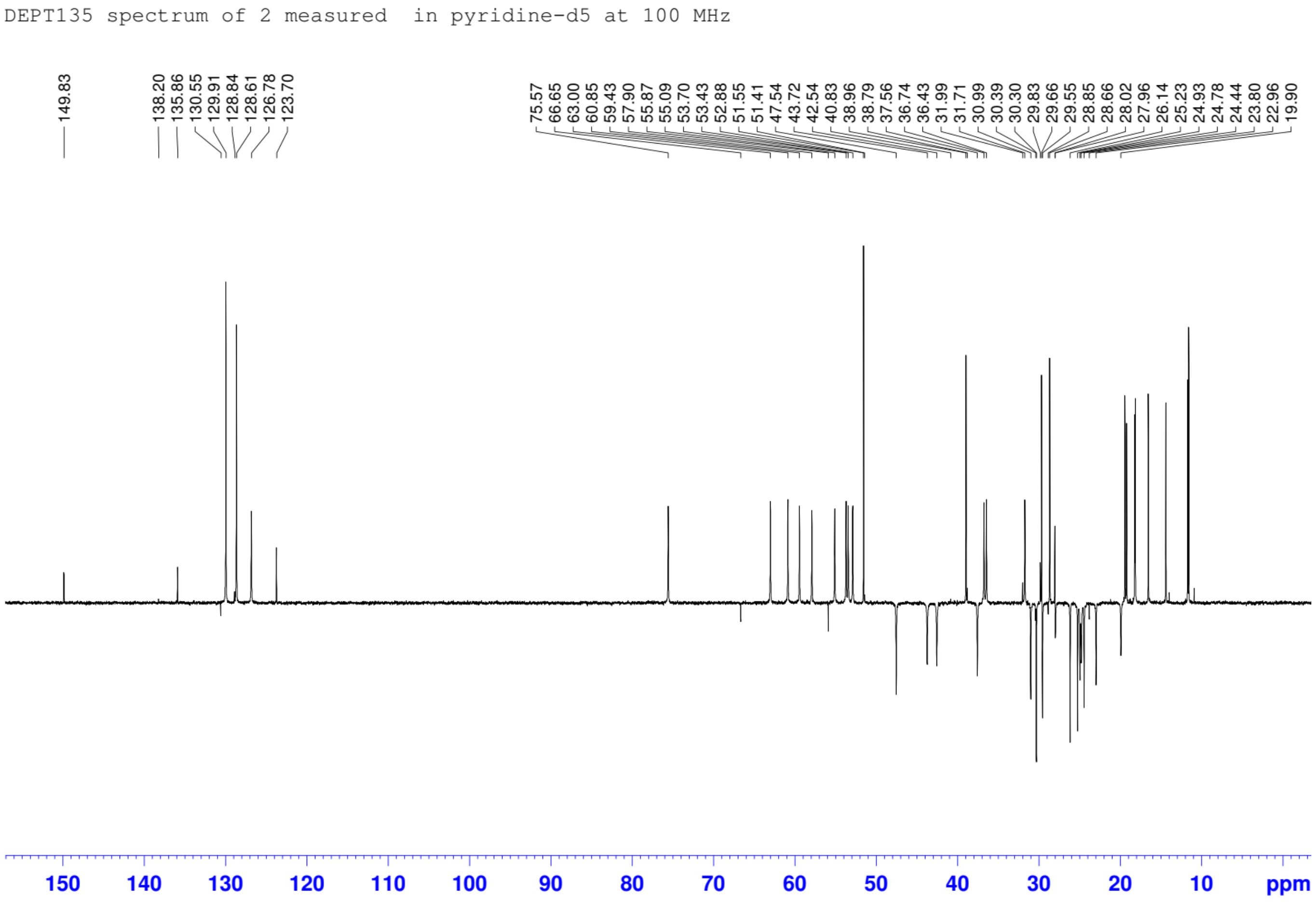

Figure S23. DEPT135 NMR spectrum of **2** in pyridine-d_5 (100 MHz).

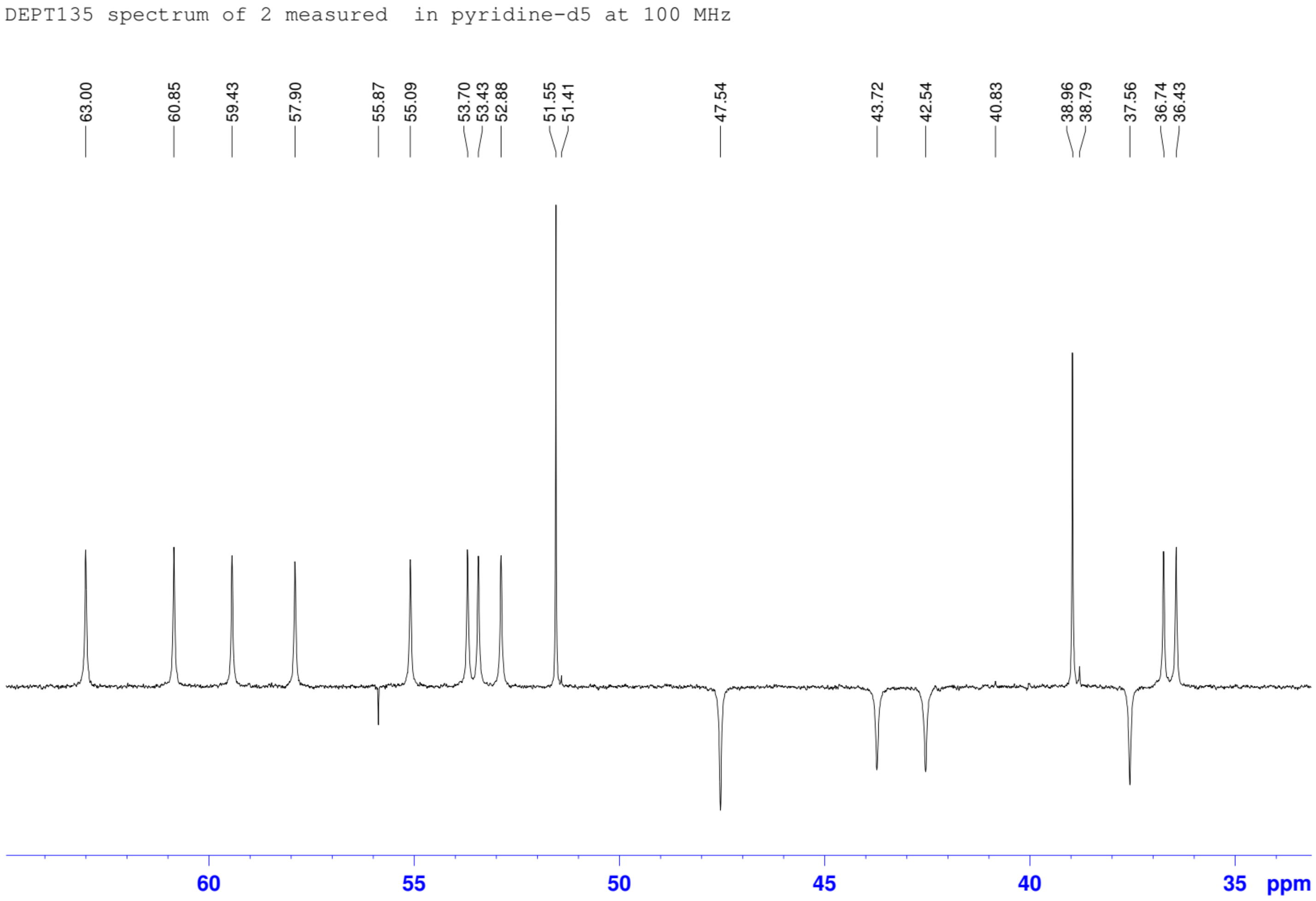

Figure S24. Enlarged DEPT135 NMR spectrum of **2** in pyridine-d_5 (100 MHz).

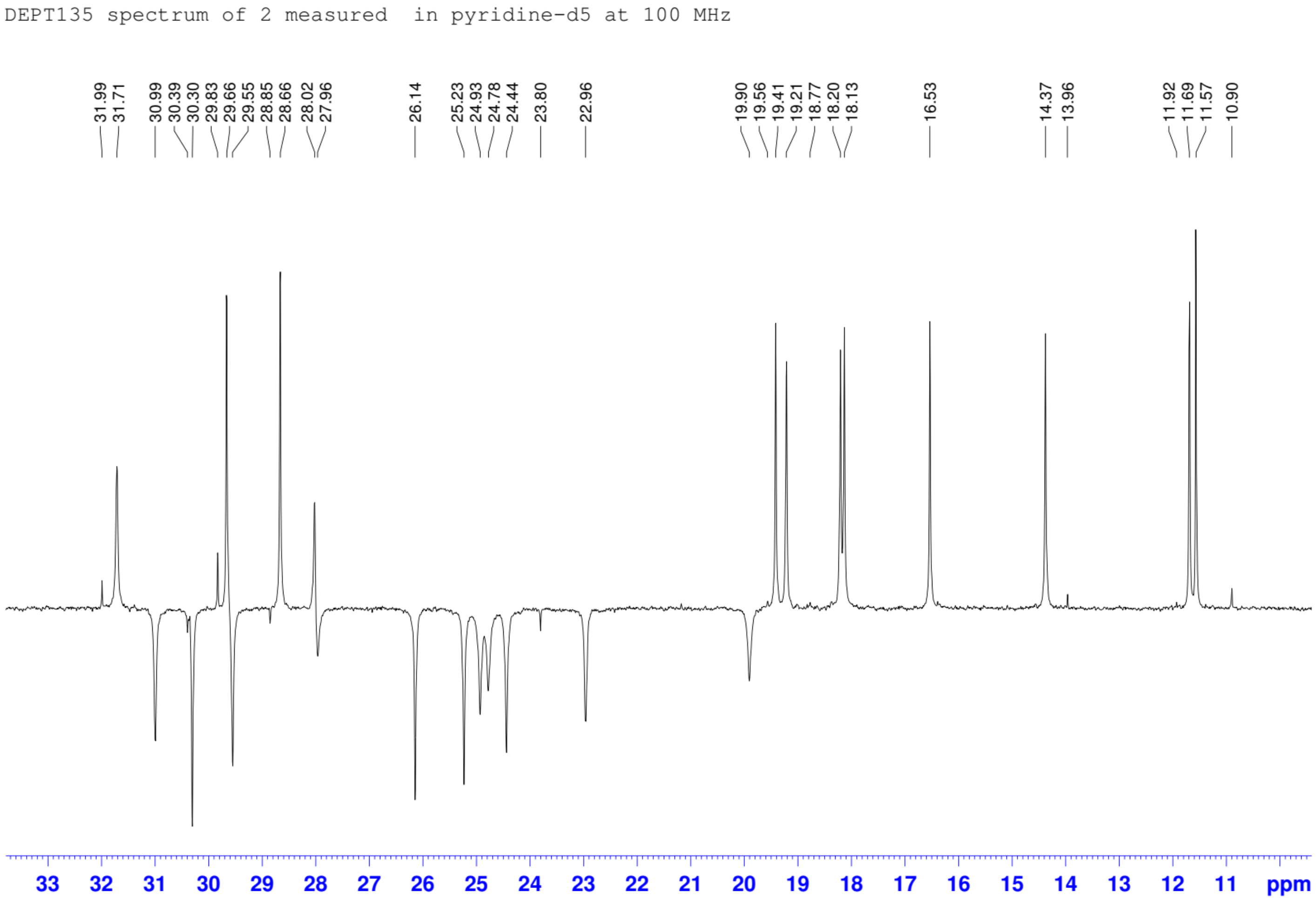

Figure S25. Enlarged DEPT135 NMR spectrum of **2** in pyridine-d_5 (100 MHz).

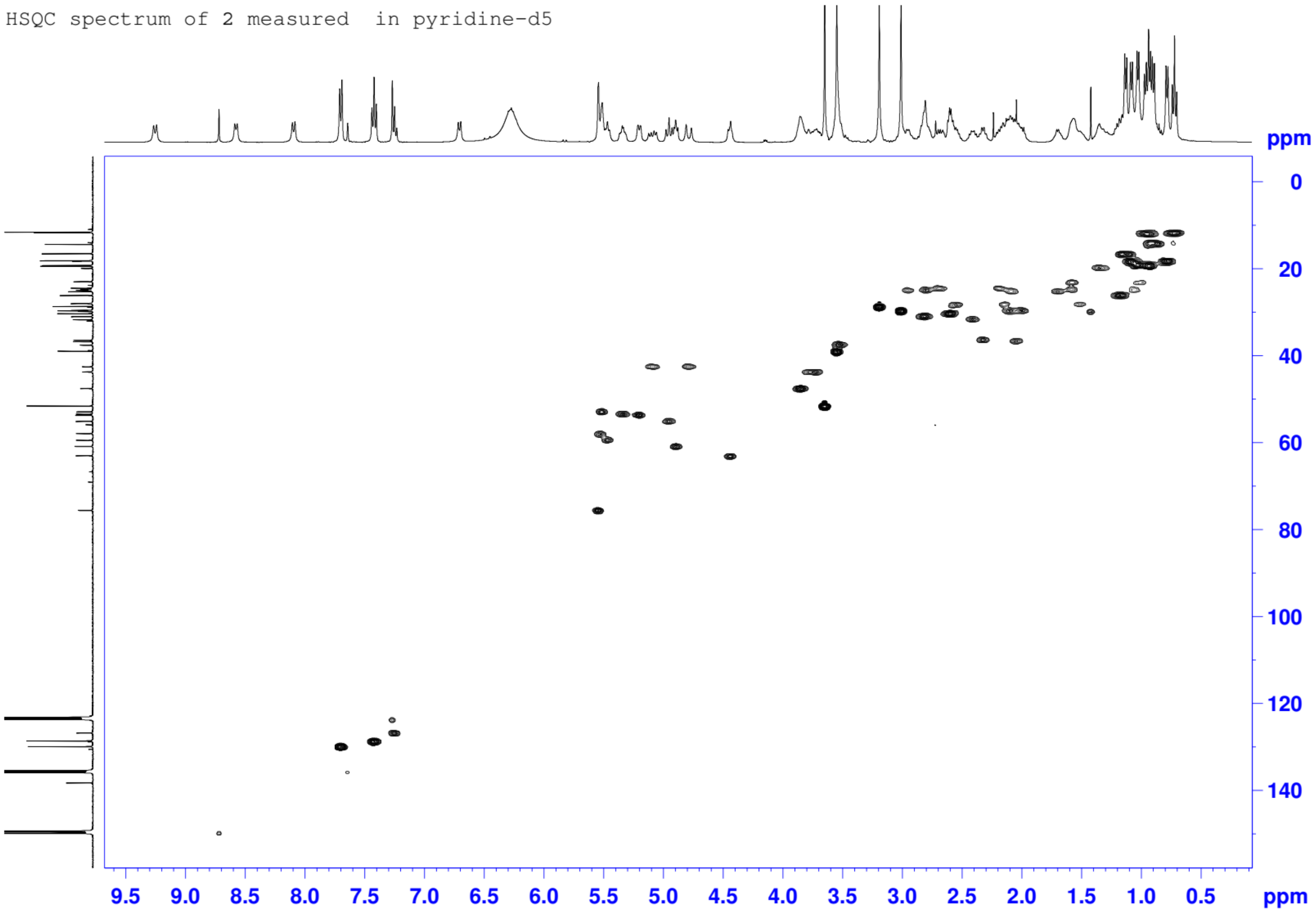

Figure S26. HSQC spectrum of **2** in pyridine-d_5.

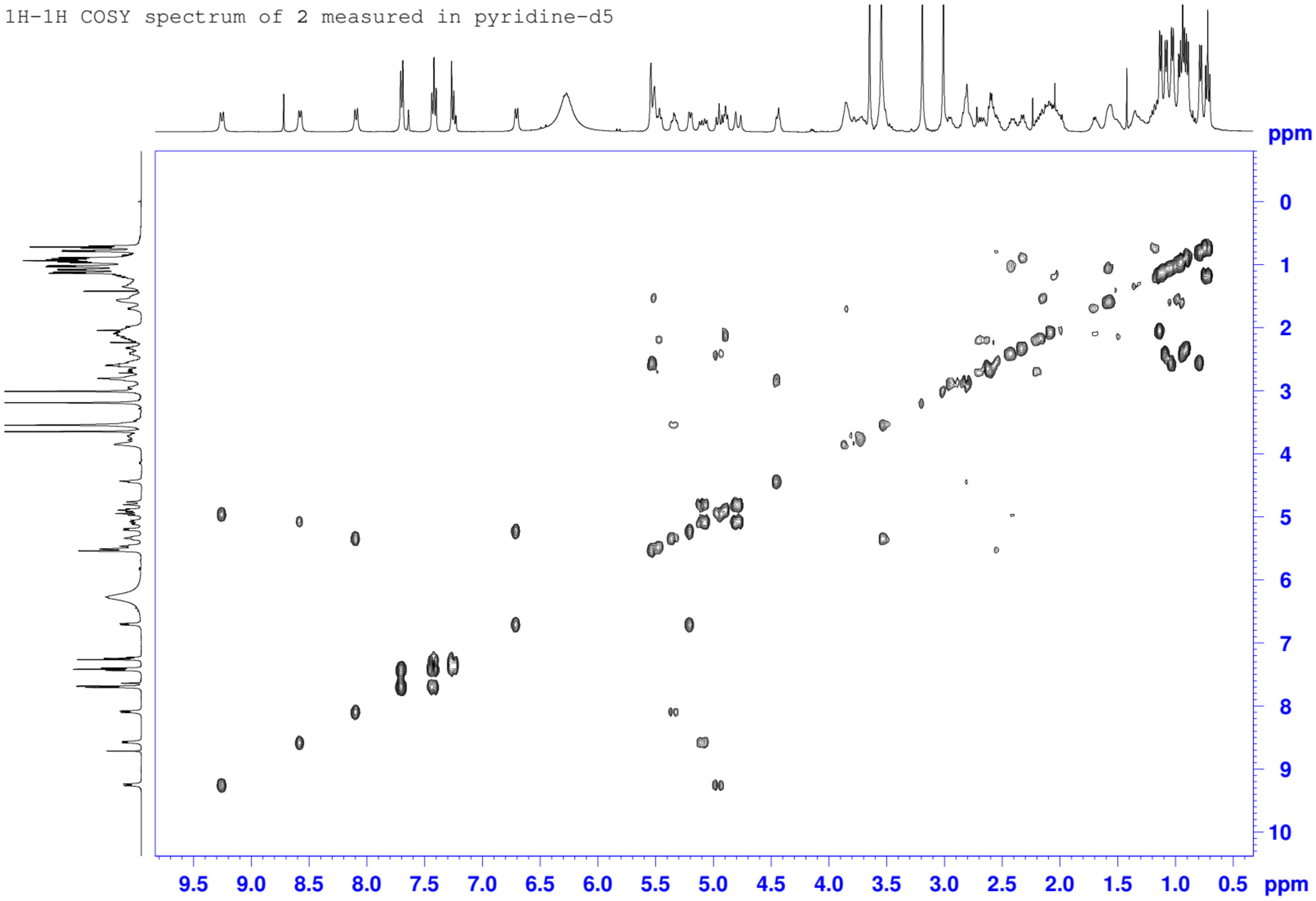

Figure S27. ^{1}H-^{1}H COSY spectrum of **2** in pyridine-d_5.

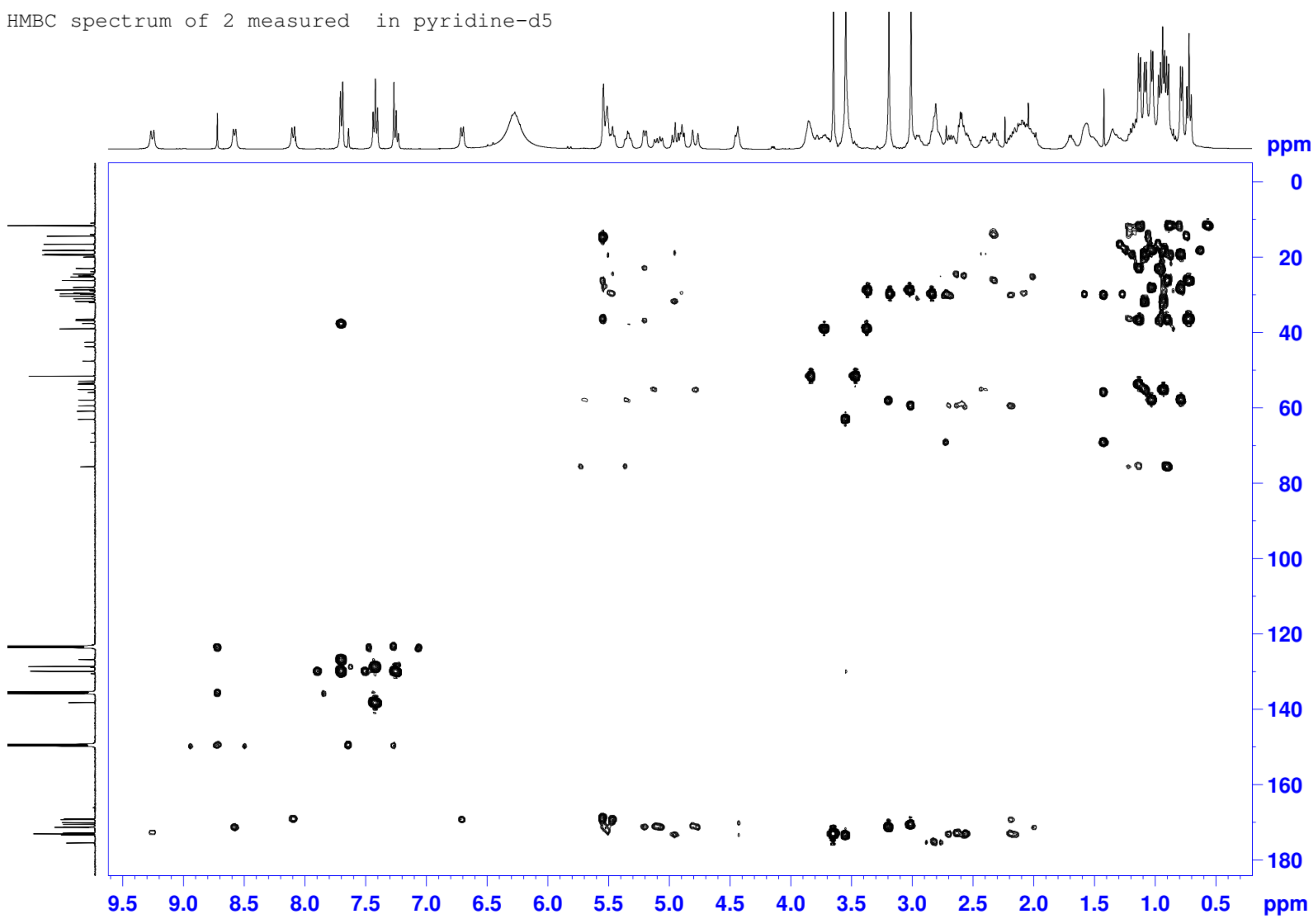

Figure S28. HMBC spectrum of **2** in pyridine-d_5.

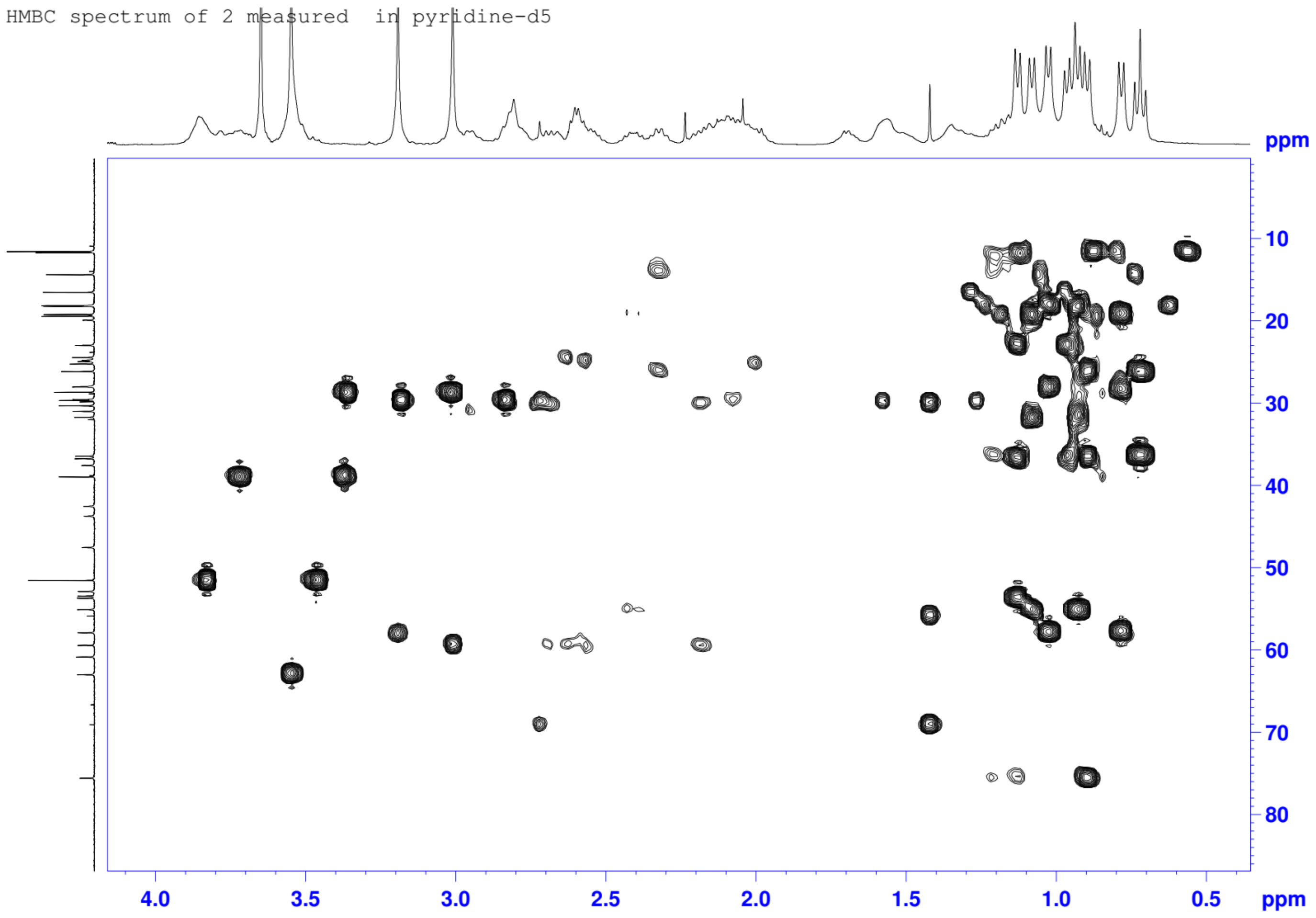

Figure S29. Enlarged HMBC spectrum of **2** in pyridine-*d*5.

HMBC spectrum of 2 measured in pyridine-d5

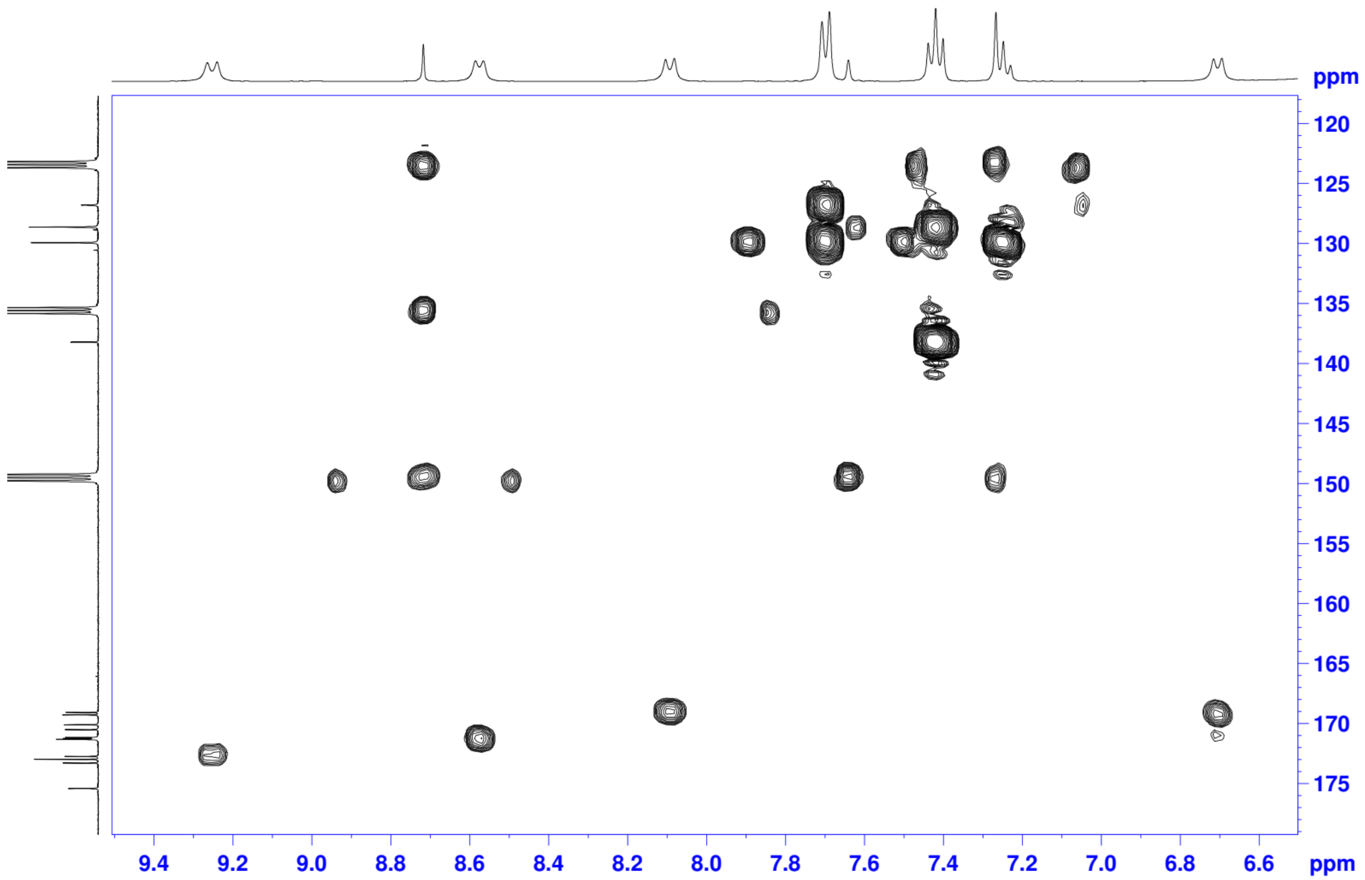

Figure S30. Enlarged HMBC spectrum of **2** in pyridine-d_5.

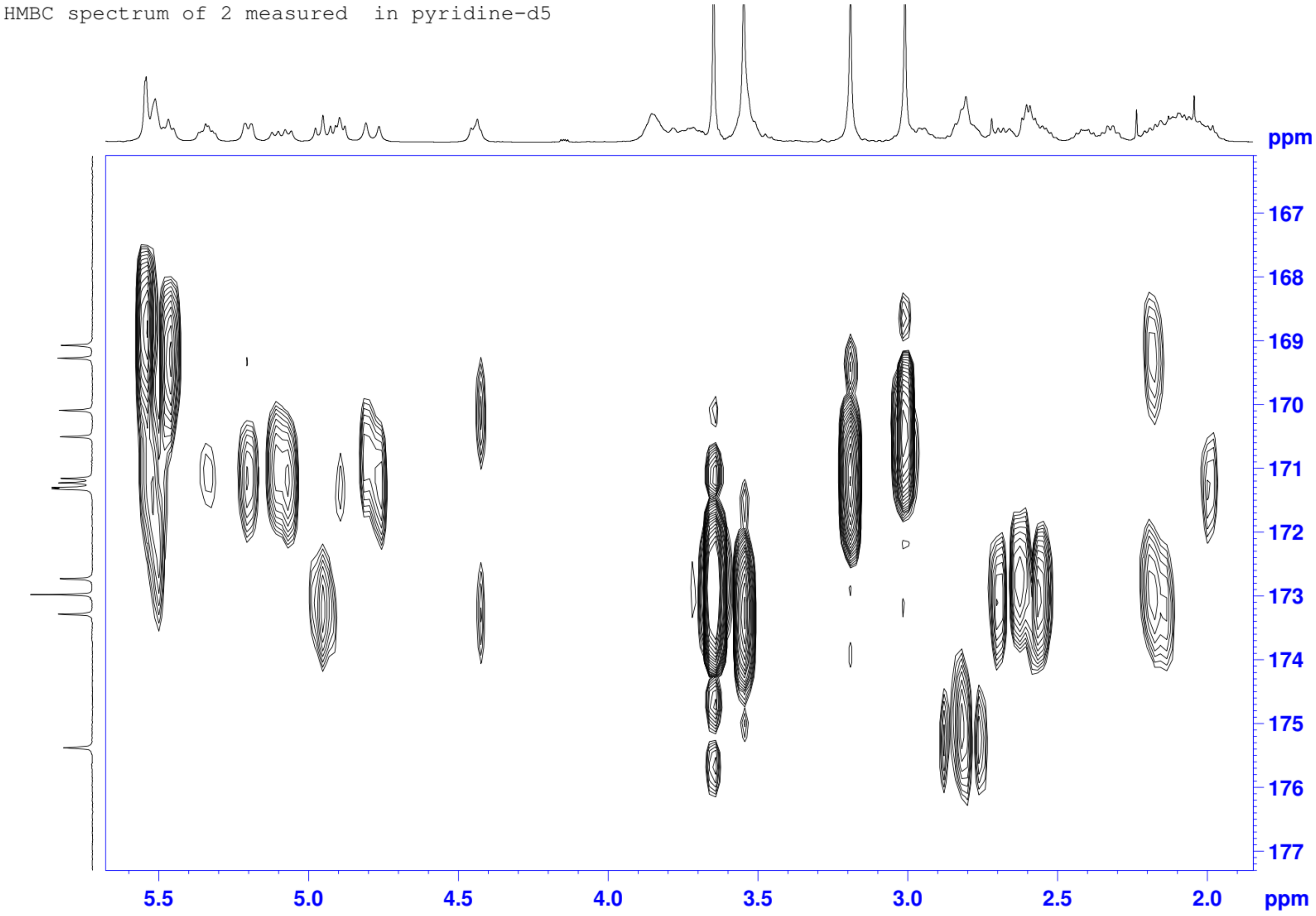

Figure S31. Enlarged HMBC spectrum of **2** in pyridine-d_5.

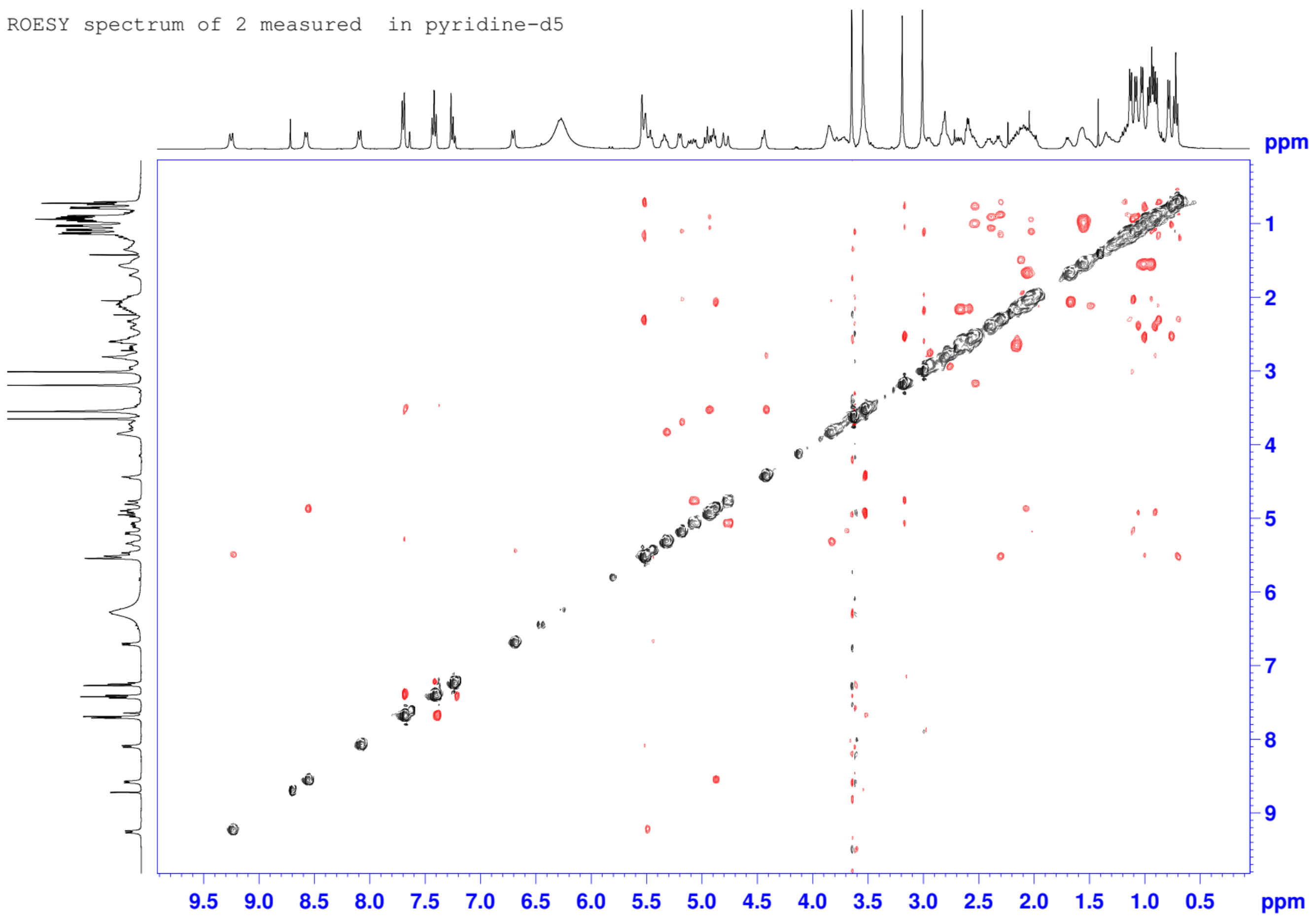

Figure S32. ROESY spectrum of **2** in pyridine-d_5.

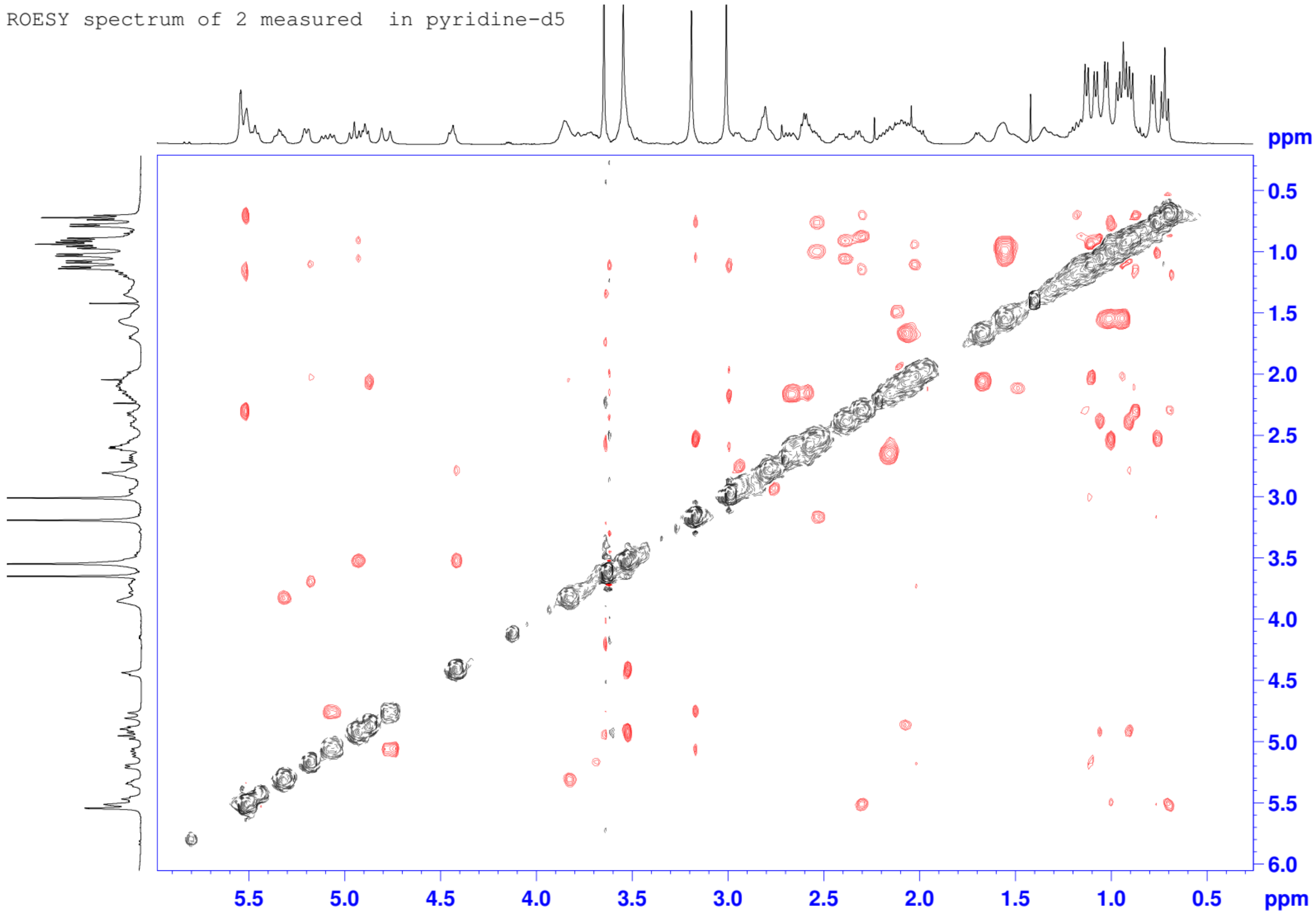

Figure S33. Enlarged ROESY spectrum of **2** in pyridine-*d*5.

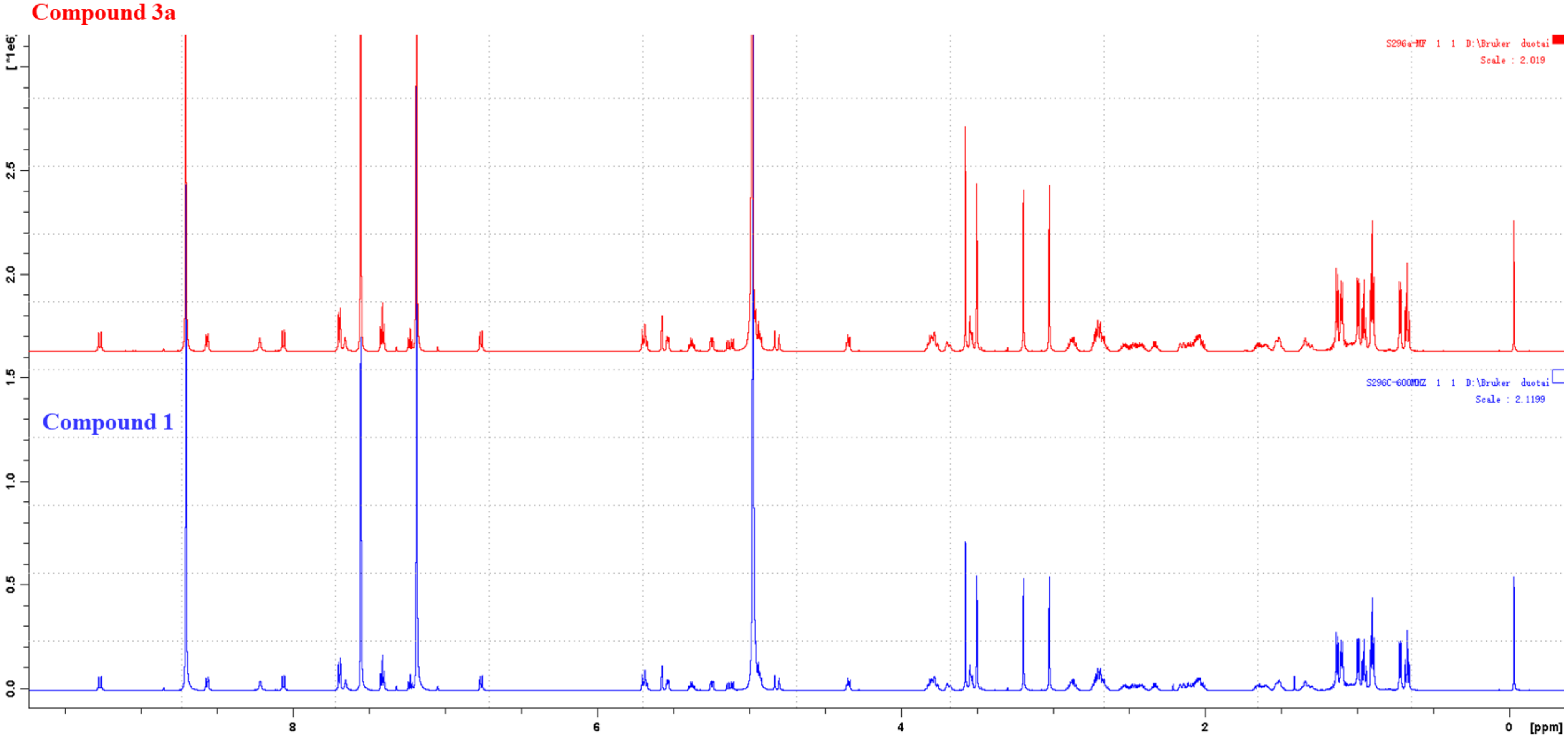

Figure S34. Comparision of ^{1}H NMR spectra of **1** and **3a** in pyridine-d_5 at 600 MHz

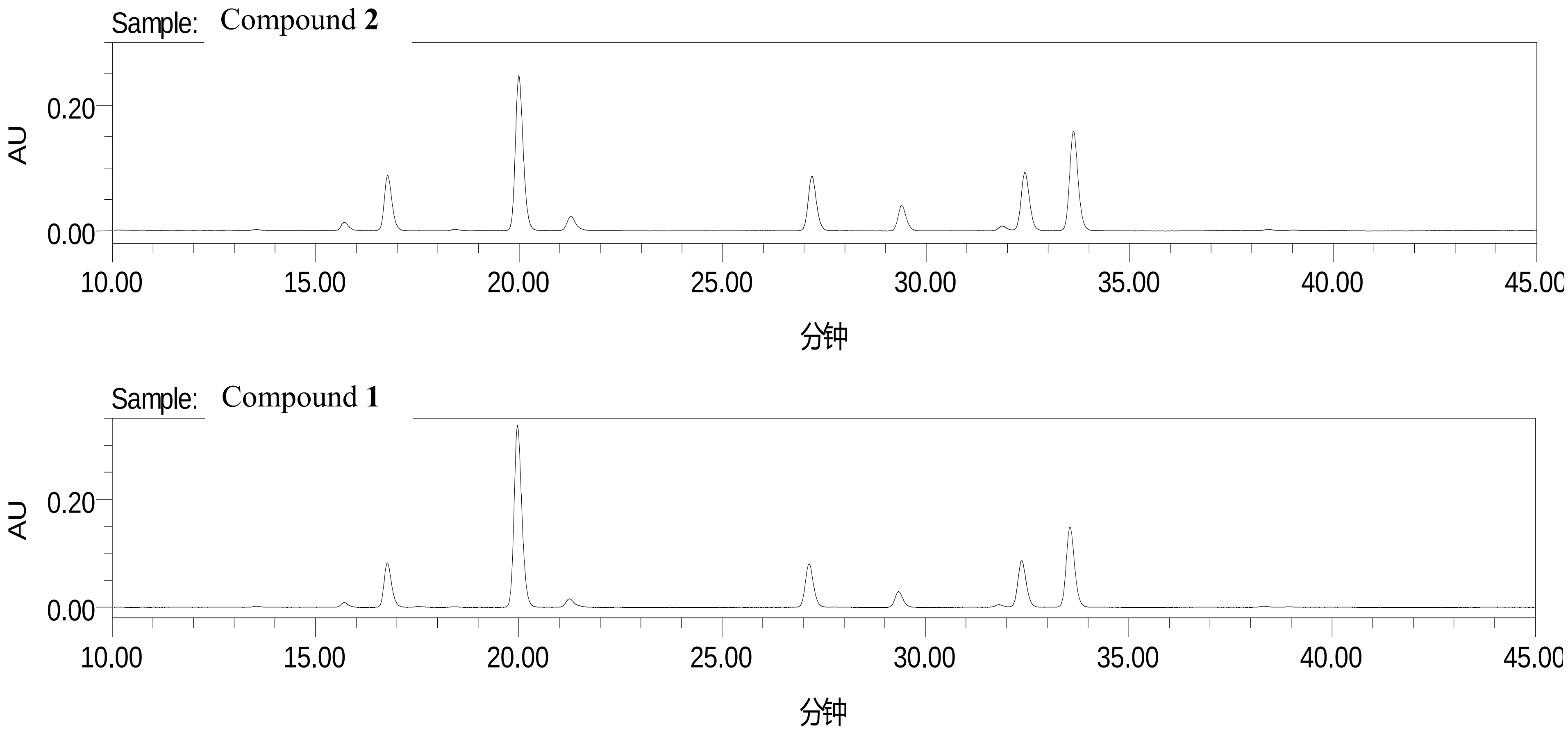

Figure S35. HPLC chromatograms of the Marfey's derivatives of **2** and **1.**
